# A toolkit for costing environmental health services in healthcare facilities

**DOI:** 10.2166/washdev.2021.018

**Published:** 2021-06-04

**Authors:** Darcy M. Anderson, J. Wren Tracy, Ryan Cronk, Hayley Schram, Nikki Behnke, Jamie Bartram

**Affiliations:** a The Water Institute, Gillings School of Global Public Health, University of North Carolina at Chapel Hill, Chapel Hill, NC 27599, USA; b ICF International, Durham, NC 27713, USA; c School of Civil Engineering, University of Leeds, Leeds, UK

**Keywords:** costing, environmental health, healthcare facilities, infection prevention, water, sanitation, hygiene (WaSH)

## Abstract

Environmental health services (EHS) are critical for safe and functional healthcare facilities (HCFs). Understanding costs is important for improving and sustaining access to EHS in HCFs, yet the understanding of costs is poor and no tools exist to specifically support costing EHS in HCFs in low- and middle-income countries. We developed a toolkit to guide the following steps of costing EHS in HCFs: defining costing goals, developing and executing a data collection plan, calculating costs, and disseminating findings. The costing toolkit is divided into eight step-by-step modules with instructions, fillable worksheets, and guidance for effective data collection. It is designed for use by diverse stakeholders involved in funding, implementation, and management of EHS in HCFs and can be used by stakeholders with no prior costing experience. This paper describes the development, structure, and functionality of the toolkit; provides guidance for its application; and identifies good practices for costing, including pilot testing data collection tools and iterating the data collection process, involving diverse stakeholders, considering long-term costs, and disaggregating environmental costs in records to facilitate future costing. The toolkit itself is provided in the Supplementary Material.

## INTRODUCTION

Environmental health services (EHS) in healthcare facilities (HCFs) – such as sanitation, waste management, and cleaning – prevent the transmission of contamination from person-to-person and person-to-environment, vice versa (Anderson *et al.* 2020). EHS are critical for safe and functional HCFs. They reduce the spread of healthcare-acquired infections, encourage care-seeking among patients, and improve workplace satisfaction of healthcare workers (Pittet *et al.* 2008; Adhikari & Supakankunit 2014; Bouzid *et al.* 2018; Gamalathge *et al.* 2019; Watson *et al.* 2019). Interventions to improve EHS in HCFs are important for preventing the development of antibiotic resistance and reducing maternal and newborn mortality in low- and middle-income countries (LMICs) (Pittet *et al.* 2008; Lee *et al.* 2011; Benova *et al.* 2014; Velleman *et al.* 2014).

EHS in HCFs in LMICs have been identified as an area of urgent need to meet the Sustainable Development Goals (SDGs) by 2030 (WHO 2020). SDGs targets 6.1 and 6.2 call for universal access to water, sanitation, and hygiene. The Joint Monitoring Program (JMP) of the World Health Organization (WHO) and UNICEF monitors these targets and tracks water, sanitation, hygiene, waste management, and cleaning as EHS in HCFs (United Nations 2015; WHO/UNICEF 2018). The JMP’s 2019 report (WHO/UNICEF 2019) indicates that approximately 26% of HCFs lack basic access to water; 21% lack sanitation, and 16% lack hand hygiene facilities. Other studies indicate that 73% of HCFs in LMICs lack sterilization equipment, 39% lack handwashing soap, and 39% lack adequate infectious waste disposal (Cronk & Bartram 2018).

Inadequate financing is a barrier to improving access, and the mobilization of financial resources is impeded by poor understanding of costs. Of the countries included in the WHO’s (2017) Global Assessment of Sanitation and Drinking Water, only 22% had a financing plan in place for EHS in HCFs that was consistently implemented (WHO 2017). Few studies have specifically examined the costs of EHS in HCFs in LMICs (see, e.g., Adhikari & Supakankunit 2014; Huttinger *et al.* 2017; Tseng *et al.* 2021). Systematic reviews indicate that evidence is scarce and suffers from low-rigor costing and poor reporting (Anderson *et al.* 2021a). Moreover, few tools exist to support data collection. A 2020 model by Anderson *et al.* (2020) is the first designed specifically for costing EHS in HCFs in LMICs. The model describes the steps required for planning and costing EHS for budgeting purposes and identifies a need to develop specific tools to support data collection for each step.

In this paper, we extend Anderson *et al.*’s (2020) model by providing a toolkit to facilitate each step of the costing process. Our costing toolkit contains modules with fillable worksheets to define costing goals, develop and execute a data collection plan, calculate costs, and disseminate findings. This paper describes the development, structure, and functionality of the toolkit; provides guidance for its application; and identifies good practices for costing. The toolkit and corresponding spreadsheet template to calculate costs are provided in Supplementary Material, Files 1 and 2, respectively.

## OVERVIEW OF THE COSTING PROCESS

**Figure 1 |:**
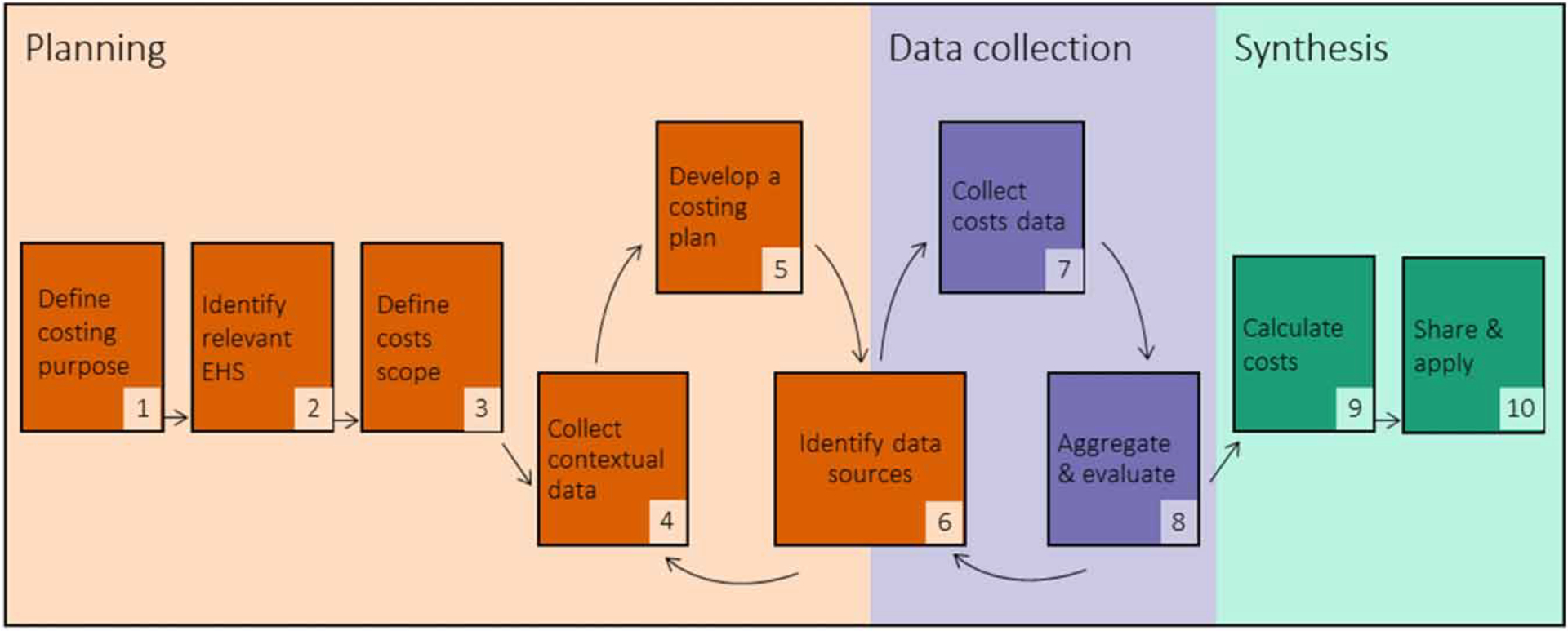
Ten steps for budgeting for EHS in HCFs. Adapted from Anderson *et al.* (2020).

In brief, Anderson *et al.*’s (2020) model describes 10 steps divided into three phases: planning, data collection, and synthesis (Figure 1). In the planning phase, stakeholders define the purpose and scope of costing, assess the facility context, develop a data collection plan, and identify data sources. In data collection, stakeholders collect, aggregate, and evaluate the completeness of costs and contextual data on EHS conditions and facility characteristics. In the synthesis phase, stakeholders calculate costs and disseminate findings. This toolkit contains eight modules to guide stakeholders through each step of the costing process, with Steps 1–3 combined into a single module.

## TOOLKIT FOR COSTING EHS IN HCFS

### Development

Initial development was informed by a review of studies cited in a systematic review on costs of EHS in HCFs (Anderson *et al.* 2021a) and Anderson *et al.*’s costing model (Anderson *et al.* 2020). We used methods described in these studies to draft tools for data collection on facility context, quantity and quality of EHS, and costs of resources necessary for EHS provision.

We tested these tools through field data collection in urban Malawi in 2018. The setting and methods are described elsewhere (Anderson *et al.* 2021b). In brief, we collected data in three individual facilities (two private clinics and a government central hospital) and a small private network of seven facilities providing care to patients enrolled in clinical research studies. We documented successes and challenges throughout data collection. We then revised tools to address these challenges and organized them into a toolkit with distinct modules for different steps of the data collection process. We documented successes as good practices for costing.

We conducted a second round of testing and revision of the compiled toolkit through field data collection in 2019 in Malawi at a rural health center and a government district hospital. We iterated our revision process as above, refining and reorganizing modules as necessary. Following the second-round testing, we also identified a need to develop a fillable spreadsheet to support costing, which we integrated into the toolkit. The resulting toolkit is presented here.

Empirical data on costs generated during toolkit development are available in Anderson *et al.* (2021b). The fillable spreadsheet in Supplementary Material, File 2 also contains example data that we collected during toolkit development.

### Target audience

We designed this toolkit for use by diverse stakeholders involved in funding, implementation, and management of EHS in HCFs. It can be used by stakeholders with no prior costing experience. We include detailed instructions for each module, worksheets to assist with the recruitment of key informants, and references for additional information when necessary.

### Outputs

Completing this toolkit in full will produce the following outputs: a list of key informants knowledgeable about EHS provision and funding; data collection plan; contextual assessment on facility characteristics (e.g., facility size, and type of services provided) and EHS characteristics (e.g., number and type of improved sanitation facilities); cost spreadsheet detailing line item expenses and associated costs; assessment of the completeness, accuracy, and limitations of data; and a dissemination plan.

These outputs may be used for planning and budgeting for EHS in HCFs, for example, by assessing the cost of operating existing EHS or predicting costs of installing new EHS. This toolkit may also be applied for research, such as for cost-effectiveness studies. Additional methods for such research (e.g., valuing benefits) are outside the scope of this paper but may be found elsewhere (Drummond *et al.* 2015; Boardman *et al.* 2017).

### Structure and functionality

The toolkit is divided into eight modules. Each module contains instructions, discussion guides, surveys, fillable worksheets, and other tools to support data collection. Table 1 describes the purpose, contents, and outputs of each module.

This toolkit is designed primarily to support bottom-up costing. Bottom-up costing is the process of enumerating all resources used for a particular EHS, then summing to calculate total costs. Modules 5–7 enumerate, assess completeness, and determine the costs of line items used in EHS delivery. The toolkit is flexible regarding the level of detail collected on individual resources. For example, costing-stakeholders may estimate costs of ‘surface cleaners’ as a broad category or enumerate and cost individual products used for surface cleaning, depending on their costing purpose.

Top-down costing estimates costs by apportioning total budgets to a given service based on some unit of analysis. For example, maintenance costs for a central sterilization department may be estimated by apportioning the total facility maintenance budget by the floor area of each department. During toolkit development, we found that expenses relevant to EHS were typically recorded in multiple departments and records systems, such that consolidated records necessary for top-down costing were not available. As such, this toolkit is not intended to support exclusively top-down costing. However, a hybrid approach that incorporates top-down costing for some items (e.g., personnel costs apportioned to an EHS from human resource salary records based on job descriptions) may be necessary. Costing-stakeholders wishing to conduct exclusively top-down costing may still find Modules 1–4 and 8 useful for planning, assessing facility context, and disseminating findings, as these modules can be applied to either top-down or bottom-up costing.

This toolkit assesses costs from the perspective of the HCF. It does not consider costs incurred by patients and caregivers (e.g., transport to the HCF) or at the health system level (e.g., advocacy). The toolkit assesses only expenses directly related to EHS provision or supervision. Indirect costs (e.g., security and administrative salaries) are not included.

### Application

We developed this toolkit for costing in individual facilities or small networks of facilities, where bottom-up costing is feasible for all EHS provision through the facility or network. The sample of HCFs in which we developed this toolkit primarily used a single modality of EHS provision per facility and had only a single unit or a small number of units where we could enumerate EHS provision in all units.

For costing large-scale provision of EHS where collecting data from all facilities is not practical, costing-stakeholders will need additional steps to identify and sample representative facilities. Similarly, this toolkit may require adaptation or additional steps for costing in large facilities with multiple units or departments, where data collection in each is not feasible. In such cases, a sampling plan for selecting representative departments may be necessary. Sampling methodology is beyond the scope of this toolkit, and we recommend that costing-stakeholders consult with sampling experts and individuals familiar with the implementation context to create a sampling plan. Once a sampling plan has been created, this toolkit can be applied in each selected facility.

Modules are designed to be completed sequentially. However, in some instances, modules may need to be repeated in whole or in part to collect all necessary data, so data collectors should be prepared to revisit and append or amend previous worksheets to ensure more comprehensive cost estimates. This toolkit is designed to be flexible to a variety of costing purposes and HCF contexts. Steps for pilot testing and adapting data collection plans and processes are included throughout the modules, and we encourage data collectors to make broader adaptations to the overall structure and content of the toolkit as necessary.

The timeline and resources required to complete this toolkit will vary based on the scope of included EHS; level of detail required; number, type, and location of facilities visited; and data sources and collection tools. Modules 1 and 3 include worksheets for identifying and aligning data collection plans with available timelines and resources.

## GOOD PRACTICES FOR COSTING

Below, we summarize good practices for costing EHS in HCFs. We base these good practices on the successes we identified during data collection for toolkit development. Box 1 provides a summary. Additional detail is provided below.

### Pilot test tools and iterate data collection

During toolkit development and application, pilot testing was critical to ensure that data collection plans were appropriate and feasible. For example, during pilot testing at two facilities, we found that questionnaires relying on recall were unreliable, as high staff turnover meant that participants were unfamiliar with long-term costs. This toolkit is designed to be flexible to a wide variety of contexts, and as such additional context-specific refining may be needed.

Fragmented funding for EHS within health systems is common (Hutton & Chase 2016), and we found the same to be true within HCFs. Given the large number of diverse goods and services required for EHS delivery, we found that information systems containing relevant data were rarely centralized. Resources required for EHS delivery were purchased by multiple departments, and costs of construction and major rehabilitations were typically recorded in separate systems from those for routine purchases of other goods and services. In some instances, relevant data may be stored externally to the facility, such as where purchases are made by donor agencies. Iterating the modules for planning and data collection can help stakeholders identify all relevant data sources and ensure that plans collecting data from each are feasible.

### Involve stakeholders from diverse roles

Knowledge of EHS and associated costs is often fragmented across staff. We found that administrative, accounting, and procurement staff were generally well versed in the supply chains and costs for goods and services to deliver EHS but had low understanding of how these resources were used in the care environment or the scope of EHS. The opposite was true for healthcare providers and clinical support staff. Knowledge of maintenance and repair processes for EHS infrastructure (e.g., toilets and sinks) was low among most staff except for dedicated maintenance workers.

Our toolkit contains modules to identify stakeholders involved in EHS provision. We recommend including stakeholders in administrative, accounting, care provision, clinical support (e.g., cleaners and waste handlers), and maintenance roles. Focus groups with stakeholders from multiple roles may prove more useful and efficient than individual interviews, particularly for the aggregation and evaluation of data, as focus groups allow for participants to receive and reflect on information from other stakeholders in real time.

### Use records to overcome poor recall

We found that high staff turnover impeded recall of infrequent expenses, particularly for repair, maintenance, and construction costs. Major repairs were infrequent, and staff with knowledge of past repair needs often were no longer employed at the facility. Even when participants were employed at the facility at the time of repairs, long recall periods meant that they could not reliably report the type or cost of repairs.

Records such as inventories and logs are valuable resources for assessing long-term costs of EHS provision. While the use of electronic health information systems in LMICs is growing, many still experience challenges with accuracy, completeness, and timeliness of information (Upadhaya *et al.* 2016; Wagenaar *et al.* 2016). In cases where record systems are not available internally to the HCF, records from supplementary sources may be used, such as from comparable facilities or local contractors.

### Consider long-term costs

At one facility we visited, staff noted that there were few maintenance costs because the facility was new, but maintenance costs were expected to rise as the facility aged. For all EHS systems, costs vary throughout their lifespan, with older facilities typically having increased costs of maintenance and repairs (Dhillon 2009). Documenting long-term costs allows costing-stakeholders to capture variation in costs over time.

Costing-stakeholders may document long-term costs in several ways. Where available, records such as maintenance logs and construction contracts can be a useful source of information covering many years. Alternatively, costing-stakeholders may sample multiple facilities of different ages to approximate costs through the lifespan of various systems, though this raises complexities of how to sample comparable facilities, select representative timepoints, and appropriately weight costs from each timepoint to estimate long-term costs. As a less complex and more efficient alternative, experienced maintenance workers or contractors may be able to estimate long-term costs for a typical facility, though these estimates may not match the specific context. Where time and resources allow, costing-stakeholders may triangulate different approaches to generate more reliable estimates.

Documenting long-term costs may also be done through prospective data collection within a single facility. Prospective documentation avoids challenges associated with record deficiencies or recall bias and has potential to be low cost if incorporated into existing information management systems. However, prospective documentation may require many years to present the full picture of long-term costs, as some capital hardware for EHS have a lifespan of several decades (e.g., 20–30 years for a medical incinerator).

### Collect and report disaggregated environmental costs

Where available, records can be a useful source of information. However, at several of our data collection sites, records were not coded or disaggregated in a way that EHS expenses could be easily extracted. Coding records that did not disaggregate environmental expenses *a priori* is time-intensive and likely infeasible for paper records first requiring digitization.

*A priori* disaggregation of environmental expenses would substantially reduce the effort required for costing. Most accounting or other health information management software will have functionality to code types of expenses so they can be automatically disaggregated, and we recommend this where possible. While electronic records facilitate more efficient costing, we recognize that expensive software for health information management may not be feasible for HCFs in low-income settings, and many lack basic access to reliable power and electricity (Cronk & Bartram 2018). *In lieu* of electronic systems, separate records for EHS, such as logs documenting average annual amount and unit price of goods and services needed, could facilitate costing and may have other useful applications, such as tracking and predicting resource needs for improved supply chain management.

While costing-stakeholders will have little influence over information management decisions for existing records, they may capitalize on learnings from using this toolkit to revise information management systems to facilitate future or ongoing data collection. Where costing-stakeholders do not have direct control over information management systems, we recommend that they debrief with administrators to describe successes and challenges of interacting with information management systems to make data collection easier for future users.

### Make findings publicly available

We encourage costing-stakeholders using this toolkit to make their findings publicly available when possible. Module 8 of this toolkit contains a guide for developing dissemination plans. Cost data can typically be made publicly available without ethical concerns related to human subjects research, provided that approval is received from facility administrators and relevant local authorities. The current evidence for costs of EHS in HCFs is poor and insufficient to estimate a range for the costs of providing basic services in different facility types (Anderson *et al.* 2021). Increasing the evidence base for costs of achieving basic and more advanced service levels across facility types can inform investment and advance progress toward targets for universal access.

## Supplementary Material

Supplemental File 1

Supplemental File 2

## Figures and Tables

**Table 1 |:** Purpose, contents, and outputs of modules in the costing toolkit

Module name	Purpose	Contents	Outputs
Introduction	Orient costing-stakeholders and facility administrators to the costing process and toolkit	Overview of toolkit structure, functionality, and contents	n/a
Module 1: preliminary planning	Define costing purpose(s); determine relevant EHS and scope of costs data to be collected	Worksheet to support preliminary planning	n/a
Module 2: key informant identification	Identify key informants to provide information on costs and facility context	Worksheets to identify key informants, including facility staff, contractors, and local partners	List of key informants to complete subsequent modules
Module 3: data collection planning	Create a plan for costs data collection, including identifying a data collection approach, location, and data sources	Guidance on selecting appropriate data collection approaches Data collection planning template Worksheet to assess costing plan feasibility	Data collection plan
Module 4: facility context assessment	Document contextual information on facility characteristics (e.g., size, patient volume, and services provided) and EHS provision	Worksheets to assess facility context indicators and environmental health conditions indicators	Contextual assessment on facility characteristics Contextual assessment on EHS characteristics
Module 5: line item identification	Identify resources used in EHS delivery; resources represent expenses to be costed in subsequent modules	Worksheets for each EHS to document resource inputs Costing spreadsheet to document resource inputs	n/a
Module 6: line item completeness evaluation	Evaluate the completeness of line items identified in Module 5 by comparing identified versus expected expenses	Frameworks of expected expenses for each EHS Worksheet to assess line item completeness using frameworks	Assessment of the completeness, accuracy, and limitations of data
Module 7: cost data collection and calculations	Collect information on the costs of line items identified in Module 5, as total costs and/or as quantities and unit costs	Guidance on developing tools (e.g., surveys and codebooks) for cost data collection Worksheets to design, pilot test, revise data collection tools; and to collect and calculate cost information	Spreadsheet documenting line item expenses and associated costs
Module 8: internal review and dissemination	Assess the information collected in previous models for accuracy, completeness, and fitness for purpose; develop a dissemination plan	Worksheet to assess data accuracy, completeness Guidance on developing dissemination plans	Dissemination plan

## Box 1 | Good practices for costing environmental health in HCFs

1. Pilot test data collection tools before beginning data collection.
2. Iterate steps of this toolkit as necessary for complete and accurate data.
3. Involve diverse staff from the HCF in data collection, including healthcare providers, cleaners, maintenance workers, logistics and procurement staff, accountants and finance staff, and administrative staff.
4. Use records (e.g., budgets, inventories, and maintenance logs) when available.
5. Consider long-term costs of operations and maintenance, which will vary as EHS systems age.
6. Disaggregate environmental expenses within information systems to facilitate future costing.
7. Make findings publicly available to build the evidence base.

## HIGHLIGHTS

- We developed a toolkit to cost environmental health services in healthcare facilities, comprising eight modules and worksheets.
- Costing-stakeholders should test tools, iterate data collection steps, involve diverse stakeholders, and consider life cycle costs.
- Costing-stakeholders should test tools, iterate data collection steps, involve diverse stakeholders, and consider long-term costs.
- Information management systems should code and disaggregate environmental costs to facilitate future costing.
