# Supplementary material for: A toolkit for costing environmental health services in healthcare facilities
Source: J Water Sanit Hyg Dev. Author manuscript; Available in PMC 2021 Sep 2. (PMC8411608; doi:10.2166/washdev.2021.018)
Supplement: Supplemental File 1 [file NIHMS1721656-supplement-Supplemental_File_1.docx]

**Toolkit for costing environmental health services in healthcare facilities**


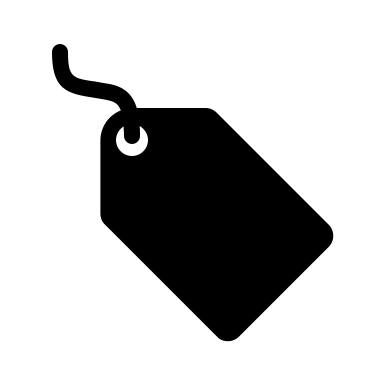

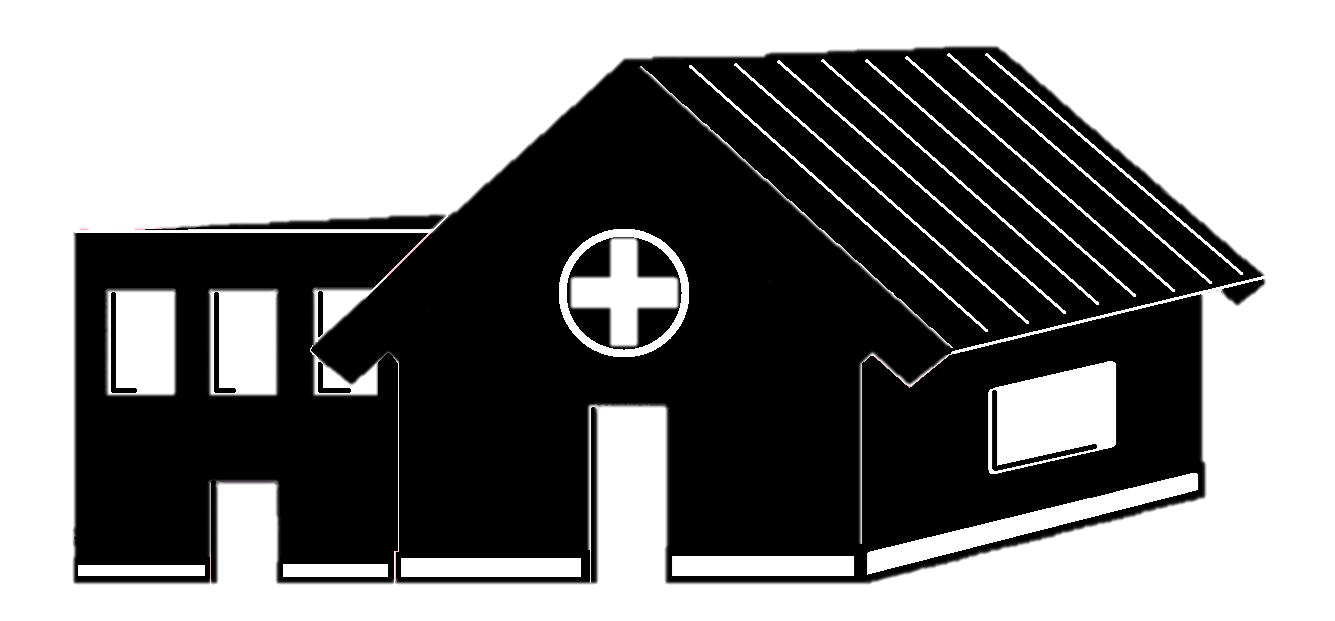


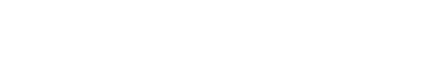


The Water Institute at UNC

Gillings School of Global Public Health

The University of North Carolina at Chapel Hill

Rosenau Hall, CB #7431

135 Dauer Drive, Chapel Hill, NC 27599-7431

Phone +1-919-966-7302

Fax +1 -919-966-7911

<http://www.waterinstitute.unc.edu>

This toolkit has been prepared by the following authors:

Darcy M. Anderson, J. Wren Tracy, Ryan Cronk, Hayley Schram, Nikki Behnke, and Jamie Bartram

Address correspondence to: [darcy.anderson@unc.edu](mailto:darcy.anderson@unc.edu)

© The University of North Carolina at Chapel Hill 2020

Contents

[Contents ii](#_Toc67313850)

[List of figures iii](#_Toc67313851)

[List of tables iii](#_Toc67313852)

[List of abbreviations iii](#_Toc67313853)

[Background iv](#_Toc67313854)

[Definitions vi](#_Toc67313855)

[References vii](#_Toc67313856)

[Introduction to the toolkit 1](#_Toc67313857)

[Module 1: Preliminary planning 1](#_Toc67313858)

[Worksheet 1.1: Planning process check 2](#_Toc67313859)

[Module 2: Key informant identification 8](#_Toc67313860)

[Worksheet 2.1: Facility informant identification 9](#_Toc67313861)

[Worksheet 2.2: Contractor and local partner identification 11](#_Toc67313862)

[Module 3: Data collection planning 13](#_Toc67313863)

[Worksheet 3.1: Guidance for planning data collection 14](#_Toc67313864)

[Worksheet 3.2: Data collection plan template 18](#_Toc67313865)

[Worksheet 3.3: Data collection plan feasibility assessment 20](#_Toc67313866)

[Module 4: Facility context assessment 21](#_Toc67313867)

[Worksheet 4.1: Facility characteristics – general 22](#_Toc67313868)

[Worksheet 4.1: Facility characteristics – environmental health 25](#_Toc67313869)

[Module 5: Line item identification 35](#_Toc67313870)

[Worksheet 5.1a: Water line item identification (not piped into facility) 36](#_Toc67313871)

[Worksheet 5.1b: Water line item identification (piped into facility) 43](#_Toc67313872)

[Worksheet 5.2a: Sanitation line item identification (on site) 50](#_Toc67313873)

[Worksheet 5.2b: Sanitation line item identification (sewered) 56](#_Toc67313874)

[Worksheet 5.3: Hygiene at points of care line item identification 62](#_Toc67313875)

[Worksheet 5.4: Waste management line item identification 67](#_Toc67313876)

[Worksheet 5.5: Environmental cleaning line item identification 74](#_Toc67313877)

[Module 6: Line item completeness evaluation 79](#_Toc67313878)

[Worksheet 6.1: Identified versus expected line items comparison 80](#_Toc67313879)

[Module 7: Cost data collection and calculations 82](#_Toc67313880)

[Worksheet 7.1: Review and revise costs data collection plan, develop data collection tools 83](#_Toc67313881)

[Worksheet 7.2: Pilot test and revise data collection tools 85](#_Toc67313882)

[Worksheet 7.3: Collect and calculate costs information 86](#_Toc67313883)

[Module 8: Internal review and dissemination 88](#_Toc67313884)

[Worksheet 8.1: Internal review of findings 89](#_Toc67313885)

[Worksheet 8.2: Dissemination plan 90](#_Toc67313886)

[Appendices: frameworks of expected expenses 91](#_Toc67313887)

[Water – not piped into facility 92](#_Toc67313888)

[Water – piped into facility 93](#_Toc67313889)

[Sanitation – non-sewered 94](#_Toc67313890)

[Sanitation – sewered 95](#_Toc67313891)

[Hygiene at points of care 96](#_Toc67313892)

[Waste management 97](#_Toc67313893)

[Environmental cleaning 98](#_Toc67313894)

# List of figures

[Figure 1. Ten steps for budgeting for environmental health services (EHS) in healthcare facilities. Adapted from Anderson *et al*.^5^ v](#_Toc67315981)

[Figure 2. Joint Monitoring Program service levels for environmental health services in healthcare facilities. Available from https://washdata.org/monitoring/health-care-facilities 6](#_Toc67315982)

# List of tables

[Table 1. Contents and purpose of modules in the costing toolkit. 2](#_Toc67315983)

[Table 2. Cost categories. Reproduced from Anderson *et al*., 2020. 7](#_Toc67315984)

[Table 3. Process, prerequisites, advantages, and disadvantages for costing approaches supported by this toolkit. 15](#_Toc67315985)

# List of abbreviations

EHS Environmental health services

HCFs Healthcare facilities

JMP Joint Monitoring Program of the WHO and UNICEF

PPE Personal protective equipment

UNICEF United Nations Children’s Fund

WHO World Health Organization

Background

Maintaining environmental health services (EHS) is critical for safe, efficient care provision in healthcare facilities. Inadequate environmental conditions can reduce care seeking,^1^ efficiency and quality of care, and retention of healthcare staff;^2^ and increase risk of healthcare acquired infections.^3,4^

EHS in healthcare facilities prevent transmission of contamination from person-to-person and person-to-environment, and vice versa.^5^ EHS protect patients and healthcare providers, but also other individuals visiting or working in the facility and individuals in the surrounding community that may be exposed to waste outputs, such as dump sites. While the specific services considered to be EHS vary across disciplines, here we consider EHS monitored in healthcare facilities by The Joint Monitoring Program for Water Supply, Sanitation, and Hygiene (JMP) of the World Health Organization and UNICEF. The JMP monitors progress toward targets 6.1 and 6.2 of the Sustainable Development Goals and interprets EHS in healthcare facilities to include: water, sanitation, hygiene, healthcare waste management, and environmental cleaning.^6^

Poor understanding of the costs of EHS delivery hinders progress toward adequate provision, particularly in low and lower-middle income countries. Only 22% of countries have budgets in place for EHS in healthcare facilities that are consistently funded.^7^ Better quantification of these costs can encourage investment and facilitate improved allocation of resources for EHS in healthcare facilities.^5^

Costing is the process of collecting, recording, calculating, and reporting the costs of providing services. Cost information is critical for budgeting, which is the process of estimating and allocating resources for anticipated expenses. A process model for budgeting for environmental health services in healthcare facilities (Figure 1) has been proposed by Anderson *et al*.^5^ The model describes 10 steps across three phases: Planning, Data collection, and Synthesis.

The ten steps of the process are as follows:

1. **Define costing purpose** – Determine and agree on the purpose of costing and the intended use of costs data. The purpose of costing will dictate the type of data that must be collected and appropriate methods for data collection.
2. **Identify relevant environmental health services** – Determine which environmental health services will be the focus of costing activities, the modality/ies of provision (e.g., waste management through incineration), and the relevant provision level(s) (e.g., JMP basic level waste management).
3. **Define costs scope** – Select and justify life-cycle costs categories (see Definitions on pg. vi) and appropriate timeframes (e.g., installation only or the full life-cycle).
4. **Collect contextual data** – Contextual data are information on facility characteristics (e.g., size, patient volume, services provided); service quality and quantity; and environmental health service provision including inputs and outputs.
5. **Develop a costing plan** – Based on information from the previous steps, develop a costing plan that identifies the approach, frameworks, and costs data collection sites. Tools should be piloted and adapted as necessary.
6. **Identify data sources** – Identify key informants, departments, and records systems. Informants may be internal to the facility or external (e.g., contractors or construction firms). Assess the feasibility of the costing plan based on available data sources and revise as necessary.
7. **Collect costs data** – Execute the costing plan and collect data from all relevant sources, making sure to document each data source and iteration.
8. **Aggregate and evaluate** – Aggregate data from the various sources using the costing framework(s) developed as part of the costing plan. Compare expected versus documented costs and identify gaps. Iterate Steps 6–8 to fill any gaps.
9. **Calculate costs** – Calculate relevant unit costs and total costs, adjusting for taxes, subsidies/tariffs, financing, deprecation, or other factors.
10. **Share and apply** – Share and review results internally as a final validity check. Apply findings to budgeting practices at the facility level or share data more broadly to inform larger local or national policy changes. Plan for updating information systems, recurrent data collection, and learning.

This toolkit is a companion to this process model. This toolkit contains modules to guide you through each step, with worksheets containing discussion guides, data collection tools, and a fillable spreadsheet.


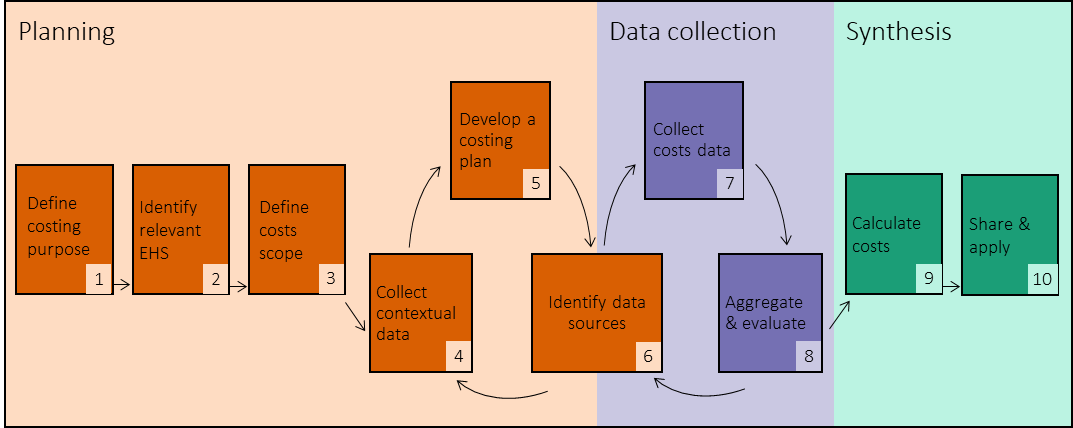


Figure 1. Ten steps for budgeting for environmental health services (EHS) in healthcare facilities. Adapted from Anderson et al.^5^

# Definitions

**Costs categories:**

**Capital hardware** – Infrastructure or equipment purchases or rentals required to establish services or implement changes to environmental health service delivery method, which are not consumed during normal environmental health service operation.

**Capital maintenance** – Expenses required to repair, rehabilitate, or otherwise maintain functionality of capital hardware, including labor costs required for these purposes.

**Capital software** – Planning, procurement, and initial training costs associated with establishing new services or implementing changes to environmental health service delivery method.

**Recurrent training** – Training required to ensure proper ongoing environmental health service provision regardless of changes to environmental health service delivery.

**Consumables** – Products and supplies that are consumed during normal operation.

**Personnel** – Labor costs associated with normal operation of a service, including staff benefits.

**Direct support** – Expenses required to supervise and monitor environmental health service provision to ensure safety and sustainability that support but do not have direct environmental health service outputs, such as auditing or developing management plans.

**Contracted services** – Fees paid to external providers to perform all or part of normal environmental health service operation, including multiple other cost categories, where expenses cannot be accurately disaggregated into categories above; where fees fall solely within another cost category described above, expenses should be included therein.

**Facility manager** – Anyone who oversees the day to day functions of the healthcare facility. This may include, but is not limited to a general administrator, the head nurse, or the head physician.

**General waste** – Waste that does not pose any particular biological, chemical, radioactive, or physical hazard.^8^

**Hygienic hand drying materials** – Any material or method of hand drying that does not pose the risk of re-contaminating the user’s hands. This includes disposable paper towels and clean cloth towels.

**Infectious waste** – Waste suspected to contain pathogens and that poses risk of disease transmission. This includes waste contaminated with blood and other body fluids as well as waste, including excreta and other materials, that have been in contact with patients infected with highly infectious diseases.^8^

**Life-cycle costing** – A costing approach that considers the lifespan of hardware from installation to operations and maintenance to decommissioning and disposal; includes both upfront installation costs and long-term operations and maintenance costs; and accounts for uneven distribution of costs over time.

**Personal protective equipment** – Any clothing or gear that is worn in order to protect the user from potential hazards. This includes, but is not limited to gloves, goggles, aprons/gowns, and masks.

**Sharps waste** – Used or unused sharps. This includes hypodermic, intravenous or other needles; auto-disable syringes; syringes with attached needles; infusion sets; scalpels; pipettes; knives; blades; broken glass.^8^

**Water quality tests**:

**Microbial** – Typically, *Escherichia coli* is used as an indicator for microbial contamination of water.

**Chemical** – Can involve testing chemical characteristics of the water such as pH, conductivity, and dissolved oxygen or testing for chemical contaminants such as arsenic, fluoride, lead, or nitrates.

**Physical** – Can involve testing of physical characteristics such as temperature and turbidity.

# References

1. Bouzid M, Cumming O, Hunter PR. What is the impact of water sanitation and hygiene in healthcare facilities on care seeking behaviour and patient satisfaction? A systematic review of the evidence from low-income and middle-income countries. *BMJ global health.* 2018;3(3):e000648.

2. D'Mello-Guyett L, Cumming O. *Water, sanitation and hygiene in health care facilities: global strategy, burden of disease, and evidence and action priorities.* London2016.

3. Pittet D, Allegranzi B, Storr J, Donaldson L. ‘Clean Care is Safer Care’: the Global Patient Safety Challenge 2005–2006. *Int. J. Infect. Dis.* 2006/11/01/ 2006;10(6):419-424.

4. Ducel G, Fabry J, Nicolle L. *Prevention of hospital-acquired infections: a practical guide.* Geneva, 2002.

5. Anderson DM, Cronk R, Best L, et al. Budgeting for environmental health services in healthcare facilities: a ten-step model for planning and costing. *Int. J. Environ. Res. Public Health.* 2020;17(6):2075.

6. WHO/UNICEF. *WASH in health care facilities: global baseline report 2019.* Geneva: WHO;2019. 9241515503.

7. WHO. *UN-Water global analysis and assessment of sanitation and drinking-water (GLAAS) 2017 report: financing universal water, sanitation and hygiene under the sustainable development goals.* Geneva: WHO; 2017.

8. Chartier Y. *Safe management of wastes from health-care activities.* World Health Organization; 2014.

Introduction to the toolkit

This toolkit will guide you through planning, collecting, and disseminating data on costs of EHS in healthcare facilities. Completing this toolkit in full will produce the following outputs:

- Data collection plan and documentation of the data collection process
- Contextual assessment on facility characteristics (e.g., facility size, type of services provided)
- Contextual assessment of environmental health characteristics (e.g., number and type of improved sanitation facilities)
- Cost spreadsheet detailing line item expenses and associated costs
- Assessment of the completeness, accuracy, and limitations of costs data
- Dissemination plan for distributing and applying costs findings

The toolkit is divided into eight modules (Table 1). Each module contains instructions, discussion guides, survey tools, fillable worksheets, and other tools to support data collection. Modules 1–3 support planning data collection. Module 4 collects contextual information about the facility and environmental health conditions. Modules 5–7 identify line items, assess their associated costs, and evaluate data completeness. Module 8 supports internal review of data accuracy and fitness for purpose and development of a dissemination plan.

As a first step before beginning any data collection, we recommend thoroughly reviewing all modules to understand their structure, contents, and purpose. Modules are sequential and are designed to be completed in order. However, in some instances, modules may need to be repeated in whole or in part to collect all necessary data, so data collectors should be prepared to revisit and append or amend previous worksheets to ensure more comprehensive costs estimates. Each module includes specific instructions for successful use.

This toolkit is designed to be flexible to a variety of costing purposes and facility contexts. Steps for pilot testing and adapting data collection plans and processes are built in throughout the modules, but we encourage data collectors to make broader adaptations to the overall structure and content of the toolkit as necessary to meet their needs. Where adaptions are made, we encourage data collectors to pilot test and document each iteration as a good practice to improve future costing.

Table 1. Contents and purpose of modules in the costing toolkit.

| **Module name** | **Purpose** | **Contents** |
| --- | --- | --- |
| Module 1:  Preliminary planning | Define costing purpose(s); determine relevant environmental health services (EHS) and scope of costs data to be collected | Worksheet to support preliminary planning |
| Module 2:  Key informant identification | Identify key informants to provide data on costs and facility context | Worksheets to identify facility informants and contractors and local partners |
| Module 3:  Data collection planning | Create a plan for costs data collection, including identifying a data collection approach, location, and data sources | Guidance on selecting appropriate data collection approaches  Data collection planning template  Worksheet to assess costing plan feasibility |
| Module 4:  Facility context assessment | Document contextual information on facility characteristics (e.g. size, patient volume, services provided) and environmental health service provision | Worksheets to assess facility context indicators and environmental health conditions indicators |
| Module 5:  Line item identification | Identify resources used in EHS delivery; resources represent expenses to be costed in subsequent modules | Worksheets for each EHS to document resource inputs  Costing spreadsheet to document resource inputs |
| Module 6:  Line item completeness evaluation | Evaluate the completeness of line items identified in Module 5 by comparing identified versus expected expenses | Frameworks of expected expenses for each EHS  Worksheet to assess line item completeness using frameworks |
| Module 7:  Cost data collection and calculations | Collect information on the costs of line items identified in Module 5, as total costs and/or as quantities and unit costs | Guidance on developing tools (e.g., surveys, codebooks) for cost data collection  Worksheets to design, pilot test, revise data collection tools; and to collect and calculate costs information |
| Module 8:  Internal review and dissemination | Assess the information collected in previous models for accuracy, completeness, and fitness for purpose; develop a dissemination plan | Worksheet to assess data accuracy, completeness  Guidance on developing dissemination plans |

This toolkit is designed primarily to support bottom-up costing. Bottom-up costing is the process of enumerating all resources used for a particular EHS, then identifying summing costs of individual resources to calculate total costs. Modules 5–7 enumerate, assess completeness, and determine costs of line items used in EHS delivery. The toolkit is flexible to the level of detail collected on individual resources. For example, data collectors may estimate costs of “surface cleaners” as a broad category or enumerate and cost individual products used for surface cleaning, depending on the costing purpose.

Top-down costing estimates costs by apportioning total budgets to a given service based on some unit of analysis. For example, costs maintenance for a central sterilization department may be estimated by apportioning the total facility maintenance budget by the floor area of each department. Expenses relevant to EHS were typically recorded in multiple departments and records systems, such that consolidated records necessary for top-down costing are often not available. As such, this toolkit is not intended to support exclusively top-down costing. However, a hybrid approach which incorporates top-down costing to cost some resources required for EHS delivery (e.g., salary costs are apportioned to an EHS from human resource salary records based on job descriptions), may be necessary.

This toolkit assesses costs from the perspective of the healthcare facility. It does not consider costs incurred by patients and caregivers (e.g., transportation to facilities) or at the health system level (e.g., advocacy). The toolkit assesses only expenses directly related to EHS provision or supervision.

Data collectors wishing to conduct exclusively top-down costing may still find Modules 1–4 and 8 useful for planning, assessing facility context, and disseminating findings, as these modules can be applied to either top-down or bottom-up costing.

Module 1: Preliminary planning

## Overview

In this module, you will define your costing goals. Defining costing goals is an important first step to ensure that data collected in subsequent modules are fit for purpose. This module will guide discussions to encourage thoughtful dialogue and careful consideration of the purpose of costing and help build clarity and consensus across stakeholders.

This module contains 1 worksheet:

- Worksheet 1.1: Planning process check

**Worksheet 1.1** contains questions to define the costing purpose, determined relevant environmental health services, and determined the scope of costs to be collected.

## Instructions

The worksheet in this module should be completed through stakeholder meetings. Relevant stakeholders include, at a minimum, members of the data collection team; individuals who will use data for decision making (e.g., policy makers); facility administrators; any relevant funding agencies; and individuals with knowledge of environmental health conditions, facility budgets, and local research ethics and administrative approvals processes. Review the contents of the worksheet before beginning, and ensure that at least one stakeholder is present with sufficient knowledge to answer each question.

Complete this module before proceeding with later modules.

# Worksheet 1.1: Planning process check

### Date of meeting:

### Stakeholders present:

### Costing Purpose:

Document responses to the following questions:

- Why are costs data being collected?
- How will costs data inform decision making?
- Will costs data inform budgets to install new services, upgrade existing inadequate services, or maintain existing adequate services?
- How much detail is needed? (e.g., Is cost of individual maintenance line items necessary, or will overall maintenance budgets suffice?)

### Target outcome(s)

Document responses to the following questions:

- What health outcomes, if any, are targeted by spending? (e.g., reduce maternal mortality)
- If health outcomes are targeted, which environmental health services will be used to improve these outcomes? What evidence exists to demonstrate that these environmental health services are effective at improving this outcome? Attach relevant evidence of effectiveness to this worksheet.
- What service delivery outcomes are targeted by spending? (e.g., provide basic sanitation)

### Relevant Environmental Health Service(s):

In the table below, indicate which environmental health service(s) will be included in costing. For all services included, document the modality of service provision (i.e., how services are provided). For example, water may be provided through on-site boreholes or municipal water service.

Document the desired level at which services will be provided. The JMP proposes four levels – no, limited, basic, and advanced service. Definitions for no, limited, and basic service are included in the table at the end of this worksheet. Definitions for advanced service are not proposed by the JMP but left to be determined at the national level by individual countries. JMP, national, or other service level definitions may be used to complete this table.

| Service | Included? | | Modality | Service Level |
| --- | --- | --- | --- | --- |
| Water | Yes | No |  |  |
| Sanitation | Yes | No |  |  |
| Hygiene | Yes | No |  |  |
| Waste Management | Yes | No |  |  |
| Environmental Cleaning | Yes | No |  |  |

### Target Facility or Facilities:

Costs data should be collected at facilities that provide environmental health services with the desired modality and service level. If the facilities where cost findings will be applied do not currently provide services at the desired level (e.g., if the purpose of costing is to install new services where none currently exist or to rehabilitate or upgrade services to higher levels), data collection for all subsequent modules will need to occur at facilities that are comparable in size and meet modality and service level targets.

Document responses to the following questions:

- Where will findings of costs data collection be applied?
- Do these facilities currently provide services at the desired level?
- Where will costs data be collected? If this location is not the same as where costs data will be applied, document that facilities are comparable and attach evidence to this worksheet.

### Scope of Relevant Costs:

In the table below, indicate which phases of the costing lifecycle will be included for each environmental service. The costing lifecycle is the full lifespan of technology, from installation to operations and maintenance, to decommissioning and disposal. Indicate the timeframe of operations and maintenance for which the service will be funded.

Indicate the cost categories which will be included. See Table 2 at the end of this worksheet for categories and definitions. Finally, indicate any phases of the lifecycle or costs categories which will be excluded from costing. Justify any exclusions.

| Lifecycle and timeframe | Cost Categories | Excluded from Scope (w/ justification) |
| --- | --- | --- |
| [Example] Install basic water service, operate and maintain for 20 years | Capital hardware, capital software, capital maintenance, consumables, personnel | Recurrent training—relevant curricula will be integrated into existing training at no extra cost |
|  |  |  |

### Available records and data sources

Document responses to the following questions:

- What financial records are kept at this facility? How can they be accessed?
- For available financial records, are environmental health service costs disaggregated or coded in such a way that they can be easily extracted?
- Does this facility have a budget specifically for environmental health? If so, what is included in this budget? If so, how can it be accessed?
- How are employment and payroll records stored in this facility? How can they be accessed?
- Does this facility keep inventory logs for consumable supplies for environmental health services? If so, how can those records be accessed?
- Does this facility keep logs of maintenance to capital hardware for environmental health services? If so, how can those records be accessed?
- Are there records of costs from when this facility was constructed that contain capital hardware and capital software costs? How can they be accessed? If not, is this information can this information be recovered elsewhere?
- Do any partners or local contractors have records of expenses that may contain relevant costs? If so, who?

### Timeline and budget for data collection activities

Document responses to the following questions:

- When will data be collected?
- What budget is available for data collection activities?

### Ethical and administrative permissions

Ensure that any necessary ethics permissions have been secured from local ethics boards. Attach a copy to this worksheet or indicate below that local ethics permission was not required. Some national or local authorities may require registration of studies. If applicable, attach a copy of study registration to this worksheet.

Secure administrative permissions from facility administrators and other relevant authorities (e.g., local health board or health ministry). Attach a copy of any administrative permissions to this worksheet. Information in this worksheet and the introductory section of this toolkit can be used to communicate with local authorities about the purpose of costing.


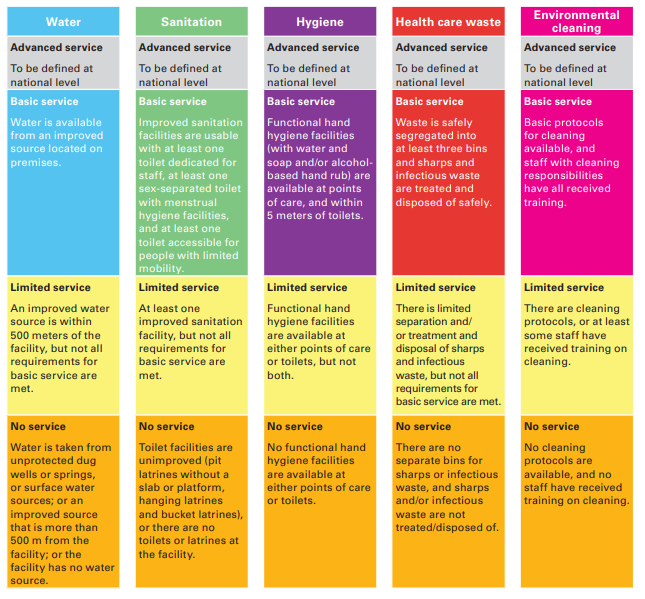


Figure 2. Joint Monitoring Program service levels for environmental health services in healthcare facilities. Available from <https://washdata.org/monitoring/health-care-facilities>

Table 2. Cost categories. Reproduced from Anderson et al., 2020.

| **Cost category** | **Definition** |
| --- | --- |
| Capital hardware | Infrastructure or equipment purchases or rentals required to establish services or implement changes to environmental health service (EHS) delivery method, which are not consumed during normal EHS operation |
| Capital maintenance | Expenses required to repair, rehabilitate, or otherwise maintain functionality of capital hardware, including labor costs required for these purposes |
| Capital software | Planning, procurement, and initial training costs associated with establishing new services or implementing changes to EHS delivery method |
| Recurrent training | Training required to ensure proper ongoing EHS provision regardless of changes to EHS delivery |
| Consumables | Products and supplies that are consumed during normal operation |
| Personnel | Labor costs associated with normal operation of a service, including staff benefits |
| Direct support | Expenses required to supervise and monitor EHS provision to ensure safety and sustainability that support but do not have direct EHS outputs, such as auditing or developing management plans |
| Contracted services | Fees paid to external providers to perform all or part of normal EHS operation, including multiple other cost categories, where expenses cannot be accurately disaggregated into categories above; where fees fall solely within another cost category described above, expenses should be included therein |

Module 2: Key informant identification

## Overview

In this module, you will identify key informants who can supply information on general facility characteristics, environmental health service provision processes, and costs.

This module contains 2 worksheets:

- Worksheet 2.1: Facility informant identification
- Worksheet 2.2: Contractor and local partner identification

**Worksheet 2.1** contains a table to document key informants within the facility. You will use this worksheet to identify informants for subsequent modules.

**Worksheet 2.2** contains a table to document key informants external to the facility. You will use this worksheet to identify informants for subsequent modules.

These worksheets may be completed in any order and should be updated as new informants are identified throughout the data collection process.

## Instructions

Worksheets in this module should be administered to a facility manager. Complete each worksheet in this section at least once, getting information from at least one manager. If after an interview, not all questions have adequate responses, contact an additional manager or a different staff member with broad knowledge of the facility for more information until there are adequate responses to all questions.

Take on the worksheets during the interview. Where possible and with the consent of the interviewee, we recommend audio-recording all interviews so that recordings may be reviewed later for clarification or to extract additional detail, if necessary.

# Worksheet 2.1: Facility informant identification

Use each question below as a prompt to fill out the table on the following page with name, title, contact information, length of employment, and knowledge area. If the length of employment for a key informant is less than six months, identify additional key informants, if available. When identifying knowledge area, the number in the table corresponds to the number of the question below. An example has been provided for reference.

1. Who can I talk to about water use, storage, testing, and treatment in this facility? List all.
2. Who can I talk to about toilet management in this facility? List all.
3. Who can I talk to about healthcare worker hygiene (i.e., handwashing and availability of alcohol-based hand rub) at points of care? List all.
4. Who can I talk to about waste management (segregation, storage, treatment, and disposal) in this facility? List all.
5. Who can I talk to about environmental cleaning in this facility? List all.
6. Who can I talk to about repairs and maintenance in this facility? List all.
7. Who can I talk to about finances and accounting in this facility? List all.
8. Who can I talk to about employment records in this facility? List all.
9. Who can I talk to about procurement and inventories of goods and services purchased in this facility? List all.

The following question does not directly correspond to a category in the table below, but instead should be asked at the end of the interview to ensure that the list of key informants is as comprehensive as possible.

Who else haven’t we identified that is important to environmental health in this facility?

**Note:** If someone is identified, ask the respondent to clarify what categories this person would be most helpful with.

Data collectors should use this worksheet throughout the data collection process, adding names as new informants continue to be identified.

Healthcare facility key informant information worksheet. Complete with Worksheet 2.1.

| Name | Title | Phone Number | Email  (if available) | Length of Employment | Knowledge Area* | | | | | | | | |
| --- | --- | --- | --- | --- | --- | --- | --- | --- | --- | --- | --- | --- | --- |
|  |  |  |  |  | 1 | 2 | 3 | 4 | 5 | 6 | 7 | 8 | 9 |
| [Example] Jane Doe | Nurse | +265 123 45 67 89 | jdoe@malawi.hcf.com | 2 years |  |  | X |  |  |  |  |  |  |
|  |  |  |  |  |  |  |  |  |  |  |  |  |  |
|  |  |  |  |  |  |  |  |  |  |  |  |  |  |
|  |  |  |  |  |  |  |  |  |  |  |  |  |  |
|  |  |  |  |  |  |  |  |  |  |  |  |  |  |
|  |  |  |  |  |  |  |  |  |  |  |  |  |  |
|  |  |  |  |  |  |  |  |  |  |  |  |  |  |
|  |  |  |  |  |  |  |  |  |  |  |  |  |  |
|  |  |  |  |  |  |  |  |  |  |  |  |  |  |
|  |  |  |  |  |  |  |  |  |  |  |  |  |  |
|  |  |  |  |  |  |  |  |  |  |  |  |  |  |
|  |  |  |  |  |  |  |  |  |  |  |  |  |  |
|  |  |  |  |  |  |  |  |  |  |  |  |  |  |
| * 1 Water use, storage, testing, and treatment; 2 Toilet management; 3 Hygiene in points of care; 4 Waste management; 5 Environmental cleaning; 6 Repairs and maintenance; 7 Finances and accounting; 8 Employment records; 9 Procurement and inventories of goods/services | | | | | | | | | | | | |  |

# Worksheet 2.2: Contractor and local partner identification

Use each question below as a prompt to fill out the table on the following page with organization name, contact name, contact title, contact information, and projects/services supported. When identifying types of projects/services the contractor or local partner support have supported, the number in the table corresponds to the number of the question below. An example has been provided for reference.

1. What contractor or local partner supports or has supported water use, storage, testing, and treatment in this facility? List all.
2. What contractor or local partner supports or has supported toilet management in this facility? List all.
3. What contractor or local partner supports or has supported hygiene (i.e., handwashing, availability of alcohol-based hand rub) at points of care in this facility? List all.
4. What contractor or local partner supports or has supported waste management (segregation, storage, treatment, and disposal) in this facility? List all.
5. What contractor or local partner supports or has supported environmental cleaning in this facility? List all.

The following question does not directly correspond to a category in the table below, but instead should be asked at the end of the interview to ensure that the list of contractors and local partners is as comprehensive as possible.

Who else haven’t we identified that is an important contractor or partner for environmental health in this facility?

**Note:** If a contractor or partner is identified, ask the respondent to clarify what categories this contractor or partner would be most helpful with.

Data collectors should use this worksheet throughout the data collection process, adding names as new contractors and local partners continue to be identified.

Healthcare facility contractor and local partner information worksheet. Complete with Worksheet 2.2

| Organization Name | Contact Name | Title | Phone Number | Email  (if available) | Projects/Services Supported | | | | |
| --- | --- | --- | --- | --- | --- | --- | --- | --- | --- |
|  |  |  |  |  | 1 | 2 | 3 | 4 | 5 |
| [Example] Waste Mgmt Malawi | John Doe | Senior Engineer | +265 123 45 43 21 | jdoe@malawi.wm.com |  |  |  | X |  |
|  |  |  |  |  |  |  |  |  |  |
|  |  |  |  |  |  |  |  |  |  |
|  |  |  |  |  |  |  |  |  |  |
|  |  |  |  |  |  |  |  |  |  |
|  |  |  |  |  |  |  |  |  |  |
|  |  |  |  |  |  |  |  |  |  |
|  |  |  |  |  |  |  |  |  |  |
|  |  |  |  |  |  |  |  |  |  |
|  |  |  |  |  |  |  |  |  |  |
|  |  |  |  |  |  |  |  |  |  |
|  |  |  |  |  |  |  |  |  |  |
|  |  |  |  |  |  |  |  |  |  |
| * 1 Water use, storage, testing, and treatment; 2 Toilet management; 3 Hygiene in points of care; 4 Waste management; 5 Environmental cleaning | | | | | | | | | |

Module 3: Data collection planning

## Overview

In this module, you will create a plan for costs data collection, including identifying a data collection approach, location, and data sources.

This module contains 3 worksheets:

- Worksheet 3.1: Guidance for planning data collection
- Worksheet 3.2: Data collection plan template
- Worksheet 3.3: Data collection plan feasibility assessment

**Worksheet 3.1** contains information to guide you through creating a data collection plan, including selecting an appropriate data collection location, approach, and data sources.

**Worksheet 3.2** contains a fillable template to document your data collection plan.

**Worksheet 3.3** contains a discussion guide to assess the feasibility and appropriateness of your data collection plan based on the costing purpose and available data sources.

These worksheets should be completed in sequence.

## Instructions

Worksheets in this module should be conducted through stakeholder meetings. Include stakeholders who participated in Module 1 and any additional key informants identified in Module 2 with knowledge on information management and records keeping practices.

These worksheets may be iterated as necessary until a feasible and appropriate data collection plan is identified.

# Worksheet 3.1: Guidance for planning data collection

Read the information provided in this worksheet in full before proceeding to Worksheet 3.2.

## Available resources and records for data collection

Resources available for data collection are an important factor in planning your data collection approach. Collecting detailed information on the quantity and price of all line items used in service provision is time and resource intensive, and data collection plans should consider this accordingly.

## Assessing quantity x unit price versus total costs

To calculate costs, you will need to answer at least one of the following questions for all resources needed to deliver the environmental health service:

- How much of each item was purchased, and at what unit cost? or
- What is the total amount spent on this item?

Collecting information on quantities and unit prices is more time and resource intensive, but gives additional information that can be applied, for example, to understand how costs may compare and scale across different facilities. You may also answer both questions for some or all cost categories or line items, and triangulate findings to compensate for the limitations of each approach.

Consult Worksheet 1.1 to determine which is more appropriate to your costing purpose, level of detail required, availability of data, and time and resources available for data collection.

## Location for data collection

If the target facilities where findings will be applied already provide services with the desired modality and service level (e.g., if the purpose of costing is to budget to maintain existing services), then data may be collected at the target facilities themselves.

However, target facilities where costs findings will be applied may not be an appropriate location for data collection. For example, if the purpose of costing is to budget for installation of environmental health services where none currently exist, data on costs must be collected from where services are provided with the desired modality and service level.

Appropriate locations for data collection may include comparable facilities or sites where similar environmental services have been installed. Worksheets in Module 4 that assess facility and environmental health services characteristics can be used to assess whether data collection locations are comparable to target facilities.

## Overview of data collection approaches

This guide is suitable for data collection through the following approaches: retrospective records review and cross-sectional interviews and surveys. An overview of the process, prerequisites, advantages, and disadvantages for each are described in Table 3. Some of the modules in this guide may be used to support prospective data collection, but the long time periods for prospective costing are beyond the scope of this toolkit.

The appropriateness of different approaches will vary based on the cost category being assessed, and multiple methods can be used for a single environmental health service. We encourage data collectors to use multiple approaches to compensate for deficiencies in individual approaches where time and resources allow.

Table 3. Process, prerequisites, advantages, and disadvantages for costing approaches supported by this toolkit.

| Approach | Process | Prerequisites | Advantages | Disadvantages |
| --- | --- | --- | --- | --- |
| Retrospective records review | Obtaining and reviewing records of spending, coding to identify relevant expenses, then summing recorded expenses to calculate total costs. | Environmental costs are included in records and are disaggregated a priori or easily identifiable  Records cover a sufficiently long time period to adequately represent purchasing patterns over the time period of interest | Efficient and accurate if comprehensive records are easily available  Does not rely on error-prone participant recall  Minimizes the time burden on facility staff | Paper-based records systems can be prohibitively time-intensive to process  Incomplete or inaccurate records can bias findings |
| Cross-sectional interviews and surveys | Recruiting knowledgeable participants to recall information on the total costs and/or quantities and unit prices of the resources used for service delivery | Availability of knowledgeable staff or external informants | Can be done in all facilities regardless of records availability | Recall of quantities and prices may be inaccurate  High-staff turn-over may limit the number of knowledgeable staff available for interview  Time intensive for data collectors and facility staff |

## Data sources

Once a costing approach has been selected, data collectors must identify appropriate data sources. For records review, consider the records that are kept internally in the facility and those that are available externally through partners, comparable facilities, contractors, suppliers, or other sources. Remember that multiple records systems may be kept within a facility, and that individual departments may maintain separate records systems. Consult Worksheets 1.1, 2.1, and 2.2 when identifying data sources.

For interviews and surveys, data sources are the participants recruited for interviews. Consult Worksheet 2.1 and 2.2 to identify relevant participants. If the participants originally contacted cannot provide the necessary information, ask if they can refer you to another participant who can.

## Timeline and budget for data collection activities

The time and budget required to apply this toolkit will vary based on the data collection approach used, scope of environmental health services included, level of detail required, and availability of suitable data sources. Retrospective records review will typically have lower upfront costs to retrieve data, particularly where data sources are electronic records. However, where records require digitization or do not disaggregate environmental expenses, the time and expense of processing and cleaning records can be substantial. In contrast, cross sectional interviews and surveys typically require more upfront costs associated with field data collection (e.g., transportation, accommodation, enumerator salaries), but allow for more targeted collection of only necessary data, reducing later expenses for data processing.

## Tips for data collection approaches and sources by cost category

Tips for collecting costs information under each cost category are provided below. These tips are not exhaustive; data collectors should use them as a starting point for costs data collection and then augment where necessary to obtain more comprehensive and robust costs data. For additional information guidance on selecting a costing approach, see the references listed at the end of this worksheet.^1–5^

- **Capital Hardware** – An invoice for installation of new infrastructure of the same design (i.e. replacement cost) could be used. External contractors, construction companies, or local implementation organizations will likely be the best sources for this information. Data collectors should additionally consider labor and installation fees.
- **Capital Maintenance** – Maintenance records, if available, could be used to determine supply needs and costs. If maintenance records are unavailable or mixed in with overall facility procurement records, data collectors may need to review hospital wide procurement records or seek costs information from the facilities preferred suppliers. Maintenance contractors may also be able to estimate an average maintenance schedule and associated costs. Data collectors should consider labor costs associated with maintenance and repairs.
- **Capital Software** – For planning and design, data collectors should seek design consultation records, where available. Where this information is not available at the facility, data collectors can identify contractors the facility would use for design consultations and procure an estimate for services. For orientations and trainings, costs should consider, where applicable, facilitator fees, equipment, materials, facilities rentals, and employee time.
- **Recurrent training** – Costs should consider, where applicable, facilitator fees, equipment, materials, facilities rentals, and employee time. Often budgets will be set for trainings, which can be a useful source of information.
- **Consumables** – Department, ward, or facility procurement records could be used. Where records track inventories of supplies but not costs, data collectors can seek costs information from the facilities preferred suppliers. Data collectors should attempt to determine the quantity used over an appropriate period of time (per day, per week, per month, etc.). Where records are lacking, costs information can be estimated through interviews of facility staff. Observations can be used to estimate quantities of goods used but are time consuming to conduct.
- **Personnel** – Given that many facilities may not track percent effort of employees, data collectors should ask key informants to estimate the percent of staff time required to perform a specific activity (e.g. percent of cleaner’s time spent cleaning toilets). Time and motion studies to observe staff activities are a more accurate but resource intensive alternative to interviews. Alternatively, where a higher level of uncertainty is acceptable in costs estimates, data collectors could include salaries of relevant staff based on job descriptions. For instance, for environmental cleaning services, it may be appropriate to allocate all of cleaners’ salaries to cleaning without regard to how much time they spend on activities.
- **Direct Support** – Salaries of supervisory personnel for direct support can use similar data sources as for personnel. Goods and services for direct support can use similar data sources as for consumables.
- **Contracted Services** – Where certain components of a service are outsourced to a contractor (e.g. waste treatment and disposal), data collectors should consult payment receipts/logs for contracted services or invoices from the associated contractor. Note that where a service is contracted, it may not be necessary to consider all the underlying costs for that service; data collectors may only need to collect data on fees paid to contractors for these services.

## References

1. Tan-Torrees Edejer T, Baltussen R, Adam T, *et al*., eds. *Making choice in health: WHO guide to cost-effectiveness analysis.* Geneva: WHO; 2003.

2. Asian Development Bank. *Guidelines for the economic analysis of projects.* Manila: World Bank Publications; 2017.

3. Drummond MF, Sculpher MJ, Claxton K, Stoddart GL, Torrance GW. *Methods for the economic evaluation of health care programmes.* Oxford University Press; 2015.

4. Anderson DM, Cronk R, Best L, *et al*. Budgeting for environmental health services in healthcare facilities: a ten-step model for planning and costing. *Int. J. Environ. Res. Public Health.* 2020;17(6):2075.

5. Chapko MK, Liu CF, Perkins M, Li YF, Fortney JC, Maciejewski ML. Equivalence of two healthcare costing methods: bottom‐up and top‐down. *Health Econ.* 2009;18(10):1188-1201.

# Worksheet 3.2: Data collection plan template

Complete the following information for each environmental health service that will be included in costing. If different approaches, locations, and/or data sources will be used for a single environmental health service, attach multiple copies of this worksheet as necessary.

### Environmental health service:

### Cost category/ies:

### Date:

### Stakeholders participating:

### Assessing quantity x unit price versus total costs

Document whether you will assess quantities and prices, total costs, or both.

### Data collection approach

Document the data collection approach that will be used.

### Data collection location

Document the location(s) where data will be collected. Specify the names of facilities where applicable.

### Data sources

Document the data sources to be used. For records, identify the specific records, their format (e.g., Excel, paper-based), and a plan for accessing them. For participants in interviews and surveys, identify specific individuals and their contact information. Consult Worksheet 2.1 and 2.2 on key informants.

## Timeline and budget for data collection activities

Based on your planned data collection approach, location, and data sources, create a timeline and budget for data collection activities. If a timeline or budget for data collection was created prior to beginning this toolkit, attach them to this worksheet.

# Worksheet 3.3: Data collection plan feasibility assessment

### Date of meeting:

### Stakeholders present:

### Costing feasibility

After completing the template in Worksheet 3.2, discuss and document your responses to the following questions. Consult Worksheet 1.1 on costing purpose and available data, as needed:

- Does the selected costing approach provide the level of detail required for the costing purpose?
- Are the time and resources allocated for data collection sufficient for the selected costing approach?
- Are the necessary data sources available and accessible at this facility? If not, can other data sources be used to supplement, such as data from contractors, comparable facilities, or local suppliers?

Based on your responses to the questions above, assess the feasibility and suitability of your data collection plan. Identify potential challenges and strategies to address them. If you determine your selected approach to be infeasible or inappropriate, revise the costing plan template in Worksheet 3.2 to align the costing purpose, target facilities, or costs scope. Document each revision and attach all revised worksheets to this module.

Module 4: Facility context assessment

## Overview

In this module, you will describe facility characteristics (e.g. size, patient volume, services provided) and document basic information on environmental health service provision. The purpose of this contextual assessment is to collect information on the quantity and quality of services provided within facilities, so that costs findings can be compared and applied across settings.

This module contains 2 worksheets:

- Worksheet 4.1: Facility Characteristics – General
- Worksheet 4.2: Facility Characteristics – Environmental Health

**Worksheet 4.1** contains a survey to record non-environmental facility characteristics related to facility size and services provided.

**Worksheet 4.2** contains a survey to basic information about the environmental health service provision modality at the facility.

These worksheets should be completed sequentially.

## Instructions

Worksheets in this section should be administered to a facility manager or other knowledgeable key informant identified in Worksheets 2.1 and 2.2. Complete each worksheet in this section at least once, getting information from at least one informant. If after an interview, not all questions have adequate responses, you will have to contact an additional informant with broad knowledge of the facility for more information until there are adequate responses to all questions.

Take on the worksheets provided. Where possible and with the consent of the interviewee, we recommend audio-recording all interviews so that recordings may be reviewed later for clarification or to extract additional detail, if necessary.

Worksheets in this module may be adapted to fit the costing purpose. If additional information on the facility context is needed, add questions to these worksheets as necessary. Worksheet 4.2 assesses environmental health service provision using JMP indicators for healthcare facilities. This worksheet could be revised to use additional or alternative indicators (e.g., compliance with national guidelines) depending on the costing purpose. However, the purpose of this assessment is to apply and compare findings across facilities, so we recommend that data collectors ensure that indicators that are widely used and accepted across areas where costs findings will be applied.

# Worksheet 4.1: Facility characteristics – general

This survey should be filled out in its entirety before moving on to the next module. Where the response to a question is “don’t know”, please identify someone in the facility who could answer the question; make sure to add this person’s contact information to the key informant worksheet, if they have not already been added.

1. Name and Title of Respondent(s):

Name Title Date of interview

1. Name of Facility:
2. Location of Facility – Region:
3. Location of Facility – District:
4. Geolocation of Facility:

Latitude:

Longitude:

1. What type of facility is this?

             Central Hospital

             District Hospital

             Health Center

             Dispensary

             Health Post

             Other (please specify):

             Don’t Know; Who would?

1. Which of the following best describes how this facility is owned?

             Public

             Private, not-for-profit

             Private, for-profit

             Public-private partnership

             Other (please specify):

             Don’t Know; Who would?

1. Is the facility open 24 hours?

             Yes

             No

             Don’t Know; Who would?

1. How many days per week is this facility routinely open?

             1

             2

             3

             4

             5

             6

             7

             Don’t Know; Who would?

1. How many total doctors/physicians/specialists work for this facility?

             Don’t Know; Who would?

1. On a normal day, how many doctors/physicians/specialists are working at a given time?

             Don’t Know; Who would?

1. How many total nurses work for this facility?

             Don’t Know; Who would?

1. On a normal day, how many nurses are working at a given time?

             Don’t Know; Who would?

1. How many total midwives work for this facility?

             Don’t Know; Who would?

1. On a normal day, how many midwives are working at a given time?

             Don’t Know; Who would?

1. How many total cleaners work for this facility?

            Don’t Know; Who would?

1. On a normal day, how many cleaners are working at a given time?

            Don’t Know; Who would?

1. How many total maintenance workers work for this facility?

            Don’t Know; Who would?

1. On a normal day, how many total maintenance workers are working at a given time?

            Don’t Know; Who would?

1. What is the total number of staff that work for this facility?

            Don’t Know; Who would?

1. On a normal day, how many total staff are working at a given time?

            Don’t Know; Who would?

1. Do you have an official record of the number of patients who receive services in this facility?

             Yes

             No

             Don’t Know; Who would?

1. Could you estimate the number of patients who receive services each day in this facility?

**Note:** This number should include any and all patients who receive services provided by the facility each day.

              Don’t Know; Who would?

1. Does this facility provide maternity services, including deliveries?

              Yes

If yes, how many deliveries occur per week?

              No

             Don’t Know; Who would?

1. How many examination rooms or points of care does this facility have?

              Don’t Know; Who would?

1. How many beds does this facility have?

              Don’t Know; Who would?

1. If the facility has inpatient beds, what is the typical bed occupancy rate?

              Don’t Know; Who would?

1. What is the annual operating budget of this facility?

              Don’t Know; Who would?

# Worksheet 4.1: Facility characteristics – environmental health

This survey should be filled out in its entirety before moving on to the next module, so where the response to a question is “don’t know”, please identify someone in the facility who could answer the question; make sure to add this person’s contact information to the key informant worksheet, if they have not already been added.

This survey can be asked to the same respondent from the General Facility Characteristics survey or can be broken up by environmental health services and asked to respondents (identified through the key informant worksheet) familiar with each respective environmental health service.

While conducting this survey, data collectors should take care to photograph water points, toilets, and points of care as well as waste management and cleaning services, making sure to document relevant aspects of each environmental health service for reference later when collecting costs information.

## Water assessment

Ask the respondent to show you to the main water supply for this facility. Then complete following questions through observation.

1. What is the main water supply type for this facility? (Choose One)

**Note:** If there is more than one source, the one used most frequently should be selected. If patients need to bring water from home because water is not available at the facility, “no water source” should be selected.

             Piped supply inside the building

             Piped supply outside the building

             Tube well / Borehole

             Protected dug well

             Unprotected dug well

             Protected spring

             Unprotected spring

             Rainwater collection

             Water-selling cart or truck

             Bottled water or sachet water

             Surface water (river/dam/lake/pond)

             Other (please specify):

             No water source

1. How far is the main water supply from the facility?

**Note:** On premises means within the building or on facility grounds. This question refers to the location from where the water is accessed for use in the health facility (e.g. tap, borehole), rather than the source where it originates.

             On premises

             Within 500 m (Less than ~ 655 steps)

             Further than 500 m (Greater than ~ 655 steps)

1. Is water available from the main water supply right now?

**Note:** Observe that water from this source is available by running or pumping the water.

             Yes

             No

## Sanitation assessment

Ask the respondent to show you a representative sample of toilets in the facility. We recommend considering the following to select a representative sample: sampling at least one toilet from every ward; sampling the most commonly used toilets in the facility; sampling at least one female-only toilet, if available; sampling at least one staff-only toilet, if available.

1. Fill out the table below and answer the following questions.

             Total number of toilets in this facility

             Total number of functional toilets in this facility (i.e., the toilet is not broken, the toilet hole is not blocked, and water is available for flush/pour-flush toilets)

             No toilet in this facility

For toilet type, indicate one of the following: Flush/Pour-flush toilet to sewer connection; Flush/Pour-flush to tank or pit; Pit latrine with slab; Composting toilet; Flush/pour-flush toilet to open drain; Pit latrine without slab/open pit; Bucket; Hanging toilet; other. If other, please describe.

|  | Toilet Type | Is it on premises? | | Does it have doors that can be locked from the inside? | | Is the door unlocked or is there a key available at all times? | | Is the hole or pit clear and unblocked? | | If it is a flush/pour flush toilet, is water available? | | Are there cracks or leaks in the toilet structure? | | Does the superstructure have any large gaps/holes? | | Does it have a private way to dispose of MHM materials? | | Are water and soap available in a private space for washing? | | Is there a sink/basin with running water within 5 meters? | | Is there soap present at the sink/basin? | | Is there alcohol-based hand rub within 5 meters? | | Can it be accessed without stairs or steps? | | Are there handrails for support on either the floor or sidewalls? | | Is the door/entryway at least 80 cm wide? | | Are there door handles and a seat within reach of people using wheelchairs or crutches? | |
| --- | --- | --- | --- | --- | --- | --- | --- | --- | --- | --- | --- | --- | --- | --- | --- | --- | --- | --- | --- | --- | --- | --- | --- | --- | --- | --- | --- | --- | --- | --- | --- | --- | --- |
| Ex | [Example]  Pit Latrine with Slab | Y | N | Y | N | Y | N | Y | N | Y | N | Y | N | Y | N | Y | N | Y | N | Y | N | Y | N | Y | N | Y | N | Y | N | Y | N | Y | N |
| 1 | N/A  No door  No door | Y | N | Y | N | Y | N | Y | N | Y | N | Y | N | Y | N | Y | N | Y | N | Y | N | Y | N | Y | N | Y | N | Y | N | Y | N | Y | N |
| 2 | N/A  No door  N/A | Y | N | Y | N | Y | N | Y | N | Y | N | Y | N | Y | N | Y | N | Y | N | Y | N | Y | N | Y | N | Y | N | Y | N | Y | N | Y | N |

|  | Toilet Type | Is it on premises? | | Does it have doors that can be locked from the inside? | | Is the door unlocked or is there a key available at all times? | | Is the hole or pit blocked? | | If it is a flush/pour flush toilet, is water available? | | Are there cracks or leaks in the toilet structure? | | Does the superstructure have any large gaps/holes? | | Does it have a private way to dispose of MHM materials? | | Are water and soap available in a private space for washing? | | Is there a sink/basin with running water within 5 meters? | | Is there soap present at the sink/basin? | | Is there alcohol-based hand rub within 5 meters? | | Can it be accessed without stairs or steps? | | Are there handrails for support on either the floor or sidewalls? | | Is the door/entryway at least 80 cm wide? | | Are there door handles and a seat within reach of people using wheelchairs or crutches? | |
| --- | --- | --- | --- | --- | --- | --- | --- | --- | --- | --- | --- | --- | --- | --- | --- | --- | --- | --- | --- | --- | --- | --- | --- | --- | --- | --- | --- | --- | --- | --- | --- | --- | --- |
| 3 | No door  No door  No door  No door  No door | Y | N | Y | N | Y | N | Y | N | Y | N | Y | N | Y | N | Y | N | Y | N | Y | N | Y | N | Y | N | Y | N | Y | N | Y | N | Y | N |
| 4 | N/A  N/A  N/A | Y | N | Y | N | Y | N | Y | N | Y | N | Y | N | Y | N | Y | N | Y | N | Y | N | Y | N | Y | N | Y | N | Y | N | Y | N | Y | N |
| 5 | N/A  N/A  N/A | Y | N | Y | N | Y | N | Y | N | Y | N | Y | N | Y | N | Y | N | Y | N | Y | N | Y | N | Y | N | Y | N | Y | N | Y | N | Y | N |
| 6 |  | Y | N | Y | N | Y | N | Y | N | Y | N | Y | N | Y | N | Y | N | Y | N | Y | N | Y | N | Y | N | Y | N | Y | N | Y | N | Y | N |
| 7 |  | Y | N | Y | N | Y | N | Y | N | Y | N | Y | N | Y | N | Y | N | Y | N | Y | N | Y | N | Y | N | Y | N | Y | N | Y | N | Y | N |
| 8 | No door  No door  No door | Y | N | Y | N | Y | N | Y | N | Y | N | Y | N | Y | N | Y | N | Y | N | Y | N | Y | N | Y | N | Y | N | Y | N | Y | N | Y | N |
| 9 | N/A | Y | N | Y | N | Y | N | Y | N | Y | N | Y | N | Y | N | Y | N | Y | N | Y | N | Y | N | Y | N | Y | N | Y | N | Y | N | Y | N |
| 10 | N/A | Y | N | Y | N | Y | N | Y | N | Y | N | Y | N | Y | N | Y | N | Y | N | Y | N | Y | N | Y | N | Y | N | Y | N | Y | N | Y | N |

1. Is there at least one toilet that is dedicated for staff only?

**Note:** Staff toilets should be for the exclusive use of staff.

             Yes

             No

1. Is there at least one toilet that is sex-separated or gender-neutral?

**Note:** Toilets in rooms with multiple stalls should all be dedicated for use by either women or men. A gender-neutral room with a single toilet is considered sex-separate, as it allows women and men to use toilets separately.

             Yes

             No

1. Is there at least one toilet that has menstrual hygiene facilities?

**Note:** A toilet can be considered to have menstrual hygiene facilities if it has a bin with a lid on it for disposal of used menstrual hygiene products and water and soap available in a private space for washing.

             Yes

             No

1. Is there at least one toilet that is available that meets the needs of people with reduced mobility?

**Note:** Toilets meet the needs of people with reduced mobility if they meet the following criteria can be accessed without stairs or steps, handrails for support are attached either to the floor or sidewalls, the door is at least 80 cm wide, the toilet has a raised seat (between 40–48 cm from the floor), a backrest and the cubicle has space for circulation/maneuvering (150 x 150 cm). The sink, tap and water outside should also be accessible and the top of the sink 75 cm from the floor (with knee clearance). Switches for lights, where relevant, should also be at an accessible height (max. 120 cm).

             Yes

             No

## Hygiene at points of care assessment

Ask the respondent to show you a representative sample of exam rooms/points of care in the facility. We recommend considering the following to select a representative sample: sampling at least one exam room/point of care from every ward; sampling the most commonly used exam rooms/points of care in the facility.

1. Observe the following. Note that observations are also needed at the point of care under the next section “Waste management assessment”.

|  | Is there a sink/basin with running water? | | Is there soap present at the sink/basin? | | Are there hygienic hand drying materials* present? | | Is there alcohol-based hand rub present? | |
| --- | --- | --- | --- | --- | --- | --- | --- | --- |
| Ex | Y | N | Y | N | Y | N | Y | N |
| 1 | Y | N | Y | N | Y | N | Y | N |
| 2 | Y | N | Y | N | Y | N | Y | N |
| 3 | Y | N | Y | N | Y | N | Y | N |
| 4 | Y | N | Y | N | Y | N | Y | N |
| 5 | Y | N | Y | N | Y | N | Y | N |
| 6 | Y | N | Y | N | Y | N | Y | N |
| 7 | Y | N | Y | N | Y | N | Y | N |
| 8 | Y | N | Y | N | Y | N | Y | N |
| 9 | Y | N | Y | N | Y | N | Y | N |
| 10 | Y | N | Y | N | Y | N | Y | N |
| * Hygienic hand drying materials include disposable paper towels or clean cloth towels | | | | | | | | |

1. Is alcohol-based hand rub available to medical staff at all times while on duty?

             Yes

             No

1. Indicate all of the following personal protective equipment that are available for healthcare providers at the point of care:

_____ Sterile gloves

_____ Face masks

_____ Respirators

_____ Face shields

_____ Goggles

_____ Gowns

_____ Aprons

## Waste management assessment

Ask the respondent to show you a representative sample of exam rooms/points of care in the facility. We recommend considering the following to select a representative sample: sampling at least one exam room/point of care from every ward; sampling the most commonly used exam rooms/points of care in the facility.

1. Observe the following. Note that observations are also needed at the point of care under the preceding section “Hygiene at points of care assessment”.

|  | Is there a puncture proof, colored/labeled bin with a lid for sharps waste? | | Is there a colored/labeled bin with a lid for infectious waste? | | Is there a colored/labeled bin for general (non-infectious, non-sharps) waste? | | Is waste properly segregated following the labels on the bins? | |
| --- | --- | --- | --- | --- | --- | --- | --- | --- |
| Ex | Y | N | Y | N | Y | N | Y | N |
| 1 | Y | N | Y | N | Y | N | Y | N |
| 2 | Y | N | Y | N | Y | N | Y | N |
| 3 | Y | N | Y | N | Y | N | Y | N |
| 4 | Y | N | Y | N | Y | N | Y | N |
| 5 | Y | N | Y | N | Y | N | Y | N |
| 6 | Y | N | Y | N | Y | N | Y | N |
| 7 | Y | N | Y | N | Y | N | Y | N |
| 8 | Y | N | Y | N | Y | N | Y | N |
| 9 | Y | N | Y | N | Y | N | Y | N |
| 10 | Y | N | Y | N | Y | N | Y | N |

1. How is infectious waste produced at this facility usually treated/disposed?

             Autoclaved

             Incinerated (two-chamber, 850–1000 °C incinerator)

             Incinerated (other)

             Burning in a protected pit

             Open burning

             Not treated, but buried in lined, protected pit

             Not treated, but collected for medical waste disposal off-site

             Open dumping without treatment

             Not treated and added to general waste

             Other (please specify):

1. How does this facility usually treat/dispose of sharps waste?

             Autoclaved

             Incinerated (two-chamber, 850–1000 °C incinerator)

             Incinerated (other)

             Burning in a protected pit

             Not treated, but buried in lined, protected pit

             Not treated, but collected for medical waste disposal off-site

             Open dumping without treatment

             Open burning

             Not treated and added to general waste

             Other (please specify):

1. How does this facility usually treat/dispose of general (non-infectious, non-sharps) waste?

             Autoclaved

             Incinerated (two-chamber, 850–1000 °C incinerator)

             Incinerated (other)

             Burning in a protected pit

             Not treated, but buried in lined, protected pit

             Not treated, but collected for medical waste disposal off-site

             Open dumping without treatment

             Open burning

             Other (please specify):

## Environmental cleaning assessment

1. Is there a step-by-step cleaning protocol for:

|  | Yes | No | Not present in facility |
| --- | --- | --- | --- |
| Floors? |  |  |  |
| Walls and doors? |  |  |  |
| Low-level and high-level surfaces and shelves? |  |  |  |
| Infectious waste/blood spills? |  |  |  |
| Sinks/hand wash basins? |  |  |  |
| Toilets? |  |  |  |
| Mattresses? |  |  |  |
| Sheets/laundry? |  |  |  |
| Medical tools/supplies? |  |  |  |

1. Is there a roster or schedule specifying responsibility for cleaning tasks and frequency at which they should be performed?

             Yes

             No

1. How many staff responsible for cleaning have received training on environmental cleaning procedures?

             All

             Some

             None

             There are no staff responsible for cleaning

*Ask the following questions if facilities offer maternity and delivery services*

1. Is the delivery bed surface cleaned between every patient?

             Yes

             No

1. Does the facility provide a clean plastic or linen sheet for every birth?

             Yes

             No

If no, do patients bring their own?              Yes              No

1. Is a clean blade or pair of scissors available for cord cutting?

             Yes

             No

1. Are clean string or cord clamps available to tie the umbilical cord?

             Yes

             No

1. Does the facility provide clean towels to dry and wrap the baby?

             Yes

             No

If no, do patients bring their own?              Yes              No

1. Does the facility provide clean cloths to wrap the mother?

             Yes

             No

If no, do patients bring their own?              Yes              No

1. Are there materials for cleaning the perineum (region from anus to vulva) of the mother?

             Yes

             No

Module 5: Line item identification

## Overview

In this module, you will identify resources used in environmental health service delivery. These resources will be entered as line items in a costing spreadsheet, with subsequent modules adding additional detail on costs of line items to calculate overall service delivery costs.

This module contains 7 worksheets:

- Worksheet 5.1a: Water – not piped into facility
- Worksheet 5.1b: Water – piped into facility
- Worksheet 5.2a: Sanitation – on site
- Worksheet 5.2b: Sanitation – sewered
- Worksheet 5.3: Hygiene at points of care
- Worksheet 5.4: Waste management
- Worksheet 5.5: Environmental cleaning

**Worksheets 5.1–5.5** correspond to specific environmental health services. Each contains questions to identify line items for each cost category (i.e., capital hardware, capital software, capital maintenance, recurrent training, consumables, personnel, direct support).

Note that interview guides 5.1 and 5.2 have two different versions, version a and version b. Data collectors should be sure to use the appropriate interview guide for the services provided in the facility. For instance, at a facility with a borehole as their main water source, data collectors should use interview guide 5.1a: “Water – not piped into facility” and do not need to use interview guide 5.1b: “Water – piped into facility.” Review the interview guides and select those which are most appropriate to the facility context.

Worksheets may be completed in any order.

## Instructions

Use the interview guides in this section at least once for each environmental health service with an associated key informant identified in Module 2 Worksheets 2.1 and 2.2 that has knowledge of the environmental health service to be discussed. If more than one key informant was identified from Module 2, interview each. If a respondent identifies any new key informant during an interview, make sure to interview all persons identified as well. This process of complementary interviews ensures that data collectors obtain the most comprehensive information for each of the environmental health services in place at this healthcare facility.

In this module, you will begin entering line items into a spreadsheet, adding information in later modules to calculate costs. Before beginning the worksheets in this module, download and review the costing spreadsheet included in the supplementary information for this publication. During the interviews, you may enter information directly into the spreadsheet, or take notes separately for later data entry. Where possible and with the consent of the interviewee, we recommend audio-recording all interviews so that recordings may be reviewed later for clarification or to extract additional detail, if necessary.

# Worksheet 5.1a: Water line item identification (not piped into facility)

## Instructions

The purpose of this interview is to identify the resources used to provide water service. These resources will be entered as line items into a costing spreadsheet. Later modules will add information about quantities and costs of each item to calculate total costs.

Read this interview guide in its entirety before conducting the interview. The guide is divided into sections for the following cost categories: capital hardware, capital software, capital maintenance, recurrent training, consumables, personnel, and direct support.

Review the costing purpose and scope identified in Worksheet 1.1. You may skip sections for costs categories that were excluded from the scope of costing activities. Where services are provided by an external contractor, you may choose to skip questions that require contacting the contractor, if your costing purpose requires only the gross payments made to contractors and not a full enumeration of all line item costs.

Download the costing spreadsheet attached as a supplementary file with this guide. Throughout the guide, text written in blue text asks about resources used in service provision. Record these resources as line items under each cost category tab in the spreadsheet. You may take notes directly in the spreadsheet, or record notes separately and transfer to the spreadsheet later. Review the spreadsheet before conducting the interview.

Participants should be identified from Worksheet 2.1: Facility informant identification. Each section suggests a participant, but additional or alternative participants may be needed to fully answer all questions. You may administer each section of the guide to participants individually, or assemble participants for all participants at once to complete the guide together in a focus group.

Where possible and with the consent of the interviewee, we recommend audio-recording the interviews so that recordings may be reviewed later for clarification or to extract additional detail, if necessary.

### Capital hardware

*The following section should be completed by an administrator, environmental health officer, or clinical supervisor with knowledge of water supply. For text in blue, record the responses as line items under the Capital Hardware tab of the costing spreadsheet.*

Name of Respondent:

Position/Title of Respondent:

Phone Number of Respondent:

Email of Respondent (if available):

Date of Interview:

1. What is the main water source for this facility? Describe the infrastructure.
2. How is water distributed in the facility? Describe the infrastructure, if any, and any tools and supplies to collect and transport water.
3. How is water accessed at the point of care? Describe the infrastructure.
4. How is drinking water accessed by facility staff, patients, and caregivers? Describe the infrastructure.
5. How is water stored for emergencies? Describe the infrastructure.
6. How is water treated to be safe for drinking? For medical procedures? Describe the infrastructure.

### Capital software

*The following section should be completed by an administrator, environmental health officer, or clinical supervisor with knowledge of how water supply infrastructure was constructed or an individual who has access to relevant records of construction. For text in blue, record the responses as line items under the Capital Software tab of the costing spreadsheet.*

Name of Respondent:

Position/Title of Respondent:

Phone Number of Respondent:

Email of Respondent (if available):

Date of Interview:

1. Describe any site assessments, planning activities, or architectural design processes conducted before installing the water source.
2. Describe any site assessments, planning activities, or architectural design processes conducted before installing the emergency storage infrastructure.
3. Describe any site assessments, planning activities, or architectural design processes conducted before installing the water treatment infrastructure.

### Capital maintenance

*The following section to be completed with by an individual with knowledge of maintenance and repairs for the water system. For text in blue, record the responses as line items under the Capital Maintenance tab of the costing spreadsheet.*

Name of Respondent:

Position/Title of Respondent:

Phone Number of Respondent:

Email of Respondent (if available):

Date of Interview:

1. Is maintenance of the main water point performed in-house or by a contractor?

*If performed in-house:*

- 1. What, if any, regular maintenance (including cleaning) is performed on the main water point?
     1. What maintenance is performed daily?
     2. What maintenance is performed weekly?
     3. What maintenance is performed monthly?
     4. What maintenance is performed yearly?
     5. What major repairs, renovations, or rehabilitations are needed on the main water point over the course of its lifespan?

*If an external contractor is used:*

- 1. How are payments to the contractor made? What payments are made?
  2. What is the name of the contractor and what is their contact information?

**Note:** Add this information to the contractor and local partner identification worksheet if not already there. If detailed information on line items costs is needed, contact the contractor and ask questions 1.1.-1.2.

1. Is maintenance of the water access points at the point of care performed in-house or by a contractor?

*If performed in-house:*

- 1. What, if any, regular maintenance (including cleaning) is performed on the water access points at the point of care?
     1. What maintenance is performed daily?
     2. What maintenance is performed weekly?
     3. What maintenance is performed monthly?
     4. What maintenance is performed yearly?
     5. What major repairs, renovations, or rehabilitations are needed on the water access at the point of care over the course of their lifespan?

*If an external contractor is used:*

- 1. How are payments to the contractor made? What payments are made?
  2. What is the name of the contractor and what is their contact information?

**Note:** Add this information to the contractor and local partner identification worksheet if not already there. If detailed information on line items costs is needed, contact the contractor and ask questions 2.1–2.2.

1. Is maintenance of the drinking water access points performed in-house or by a contractor?

*If performed in-house:*

- 1. What, if any, regular maintenance (including cleaning) is performed on the drinking water access points?
     1. What maintenance is performed daily?
     2. What maintenance is performed weekly?
     3. What maintenance is performed monthly?
     4. What maintenance is performed yearly?
     5. What major repairs, renovations, or rehabilitations are needed on the drinking water access points over the course of their lifespan?

*If an external contractor is used:*

- 1. How are payments to the contractor made? What payments are made?
  2. What is the name of the contractor and what is their contact information?

**Note:** Add this information to the contractor and local partner identification worksheet if not already there. If detailed information on line items costs is needed, contact the contractor and ask questions 3.1–3.2.

1. Is maintenance of the emergency storage system performed in-house or by a contractor?

*If performed in-house:*

- 1. What, if any, regular maintenance (including cleaning) is performed on the emergency storage system?
     1. What maintenance is performed daily?
     2. What maintenance is performed weekly?
     3. What maintenance is performed monthly?
     4. What maintenance is performed yearly?
     5. What major repairs, renovations, or rehabilitations are needed on the emergency storage system over the course of its lifespan?

*If an external contractor is used:*

- 1. How are payments to the contractor made? What payments are made?
  2. What is the name of the contractor and what is their contact information?

**Note:** Add this information to the contractor and local partner identification worksheet if not already there. If detailed information on line items costs is needed, contact the contractor and ask questions 4.1–4.2.

1. Is maintenance of the water treatment system performed in-house or by a contractor?

*If performed in-house:*

- 1. What, if any, regular maintenance (including cleaning) is performed on the water treatment system?
     1. What maintenance is performed daily?
     2. What maintenance is performed weekly?
     3. What maintenance is performed monthly?
     4. What maintenance is performed yearly?
     5. What major repairs, renovations, or rehabilitations are needed on the water treatment system over the course of its lifespan?

*If an external contractor is used:*

- 1. How are payments to the contractor made? What payments are made?
  2. What is the name of the contractor and what is their contact information?

**Note:** Add this information to the contractor and local partner identification worksheet if not already there. If detailed information on line items costs is needed, contact the contractor and ask questions 4.1–4.2.

### Recurrent training

*The following section should be completed by an administrator, environmental health officer, or clinical supervisor who has knowledge of trainings. For text in blue, record the responses as line items under the Recurrent Training tab of the costing spreadsheet.*

Name of Respondent:

Position/Title of Respondent:

Phone Number of Respondent:

Email of Respondent (if available):

Date of Interview:

1. What routine training is conducted for water source safety, testing, and operation?
2. Are any other trainings conducted related to water? If so, what?

### Consumables

*The following section should be completed by an administrator, environmental health officer, or clinical supervisor with knowledge of water supply. For text in blue, record the responses as line items under the Consumables tab of the costing spreadsheet. Line items in this section should be consumable products (i.e., products and supplies that are consumed during normal operation; e.g., disposable cups). Tools, equipment, or supplies that are re-used without being consumed over the course of normal operation are considered capital hardware and should be logged under the capital hardware tab.*

Name of Respondent:

Position/Title of Respondent:

Phone Number of Respondent:

Email of Respondent (if available):

Date of Interview:

1. What water utilities are paid at this facility?
2. Who manages the main water point for this facility?

*If facility staff manage the main water point:*

- 1. How is water from the main water point treated?
     1. What products and supplies (including personal protective equipment) are used for water treatment?
  2. How is water from the main water point tested for quality?
     1. What products and supplies (including personal protective equipment) are used for water testing?

*If a contractor external to the facility, such as a government or privately owned utility or a community water committee manages the main water point:*

- 1. What payments are made to the contractor? Who makes the payments?
  2. What is the name of the system operator and what is their contact information?

**Note:** Add this information to the contractor and local partner identification worksheet if not already there. If detailed information on line items costs is needed, contact the contractor and ask questions 5.1–5.2.

1. Is water ever tested or treated again it is distributed in the facility?
   1. What products and supplies (including personal protective equipment) are used for water testing and/or treatment within the facility?
2. What products and supplies are used for water access at the point of care, including cleaning and restocking of any storage containers.
3. What products and supplies are used for drinking water access, include cups and utensils for drinking, supplies for cleaning and restocking containers.
4. What products and supplies are used for emergency storage containers, including cleaning and restocking containers?

### Personnel

*The following section should be completed by an administrator, environmental health officer, or clinical supervisor who supervises personnel. For text in blue, record the responses as line items under the Personnel tab of the costing spreadsheet. For all of the following questions, document the title/position of the person performing the task (e.g., laboratory technician, groundskeeper), not their specific name.*

Name of Respondent:

Position/Title of Respondent:

Phone Number of Respondent:

Email of Respondent (if available):

Date of Interview:

1. Who performs the following tasks:

**Note:** Document the title/position of the person performing the task (e.g., laboratory technician, groundskeeper), not their specific name.

- 1. Water testing and treatment?
  2. Collecting and transporting water to the facility?
  3. Cleaning and restocking of water containers at the point of care?
  4. Cleaning and restocking drinking water containers?
  5. Cleaning, flushing, and refilling of emergency storage containers?

1. Does anyone else perform water-related tasks? If so, what tasks? Who?

### Direct support

*The following section should be completed by an administrator, environmental health officer, or clinical supervisor who supervises water supply. For text in blue, record the responses as line items under the Direct Support tab of the costing spreadsheet. For all questions about people, document the title/position of the person performing the task (e.g., laboratory technician, groundskeeper), not their specific name.*

Name of Respondent:

Position/Title of Respondent:

Phone Number of Respondent:

Email of Respondent (if available):

Date of Interview:

1. Who supervises the following:
   1. Operation and functionality of the main water point?
   2. Water testing and treatment?
   3. Collection and transport of water to the facility?
   4. Stocking and availability of water at the point of care?
   5. Stocking and availability of drinking water containers?
   6. Stocking and availability of emergency storage containers?
2. Who is responsible for logistics and procurement of water-related supplies?
3. Does this facility have a water safe plan? If so, who develop and monitors the plan?
4. Does anyone else supervise water-related tasks? If so, what tasks? Who?
5. Are any tools, equipment, or supplies required for audits, safety monitoring, or supervision of water you have not already described? If so, please describe.

# Worksheet 5.1b: Water line item identification (piped into facility)

## Instructions

The purpose of this interview is to identify the resources used to provide water service. These resources will be entered as line items into a costing spreadsheet. Later modules will add information about quantities and costs of each item to calculate total costs.

Read this interview guide in its entirety before conducting the interview. The guide is divided into sections for the following cost categories: capital hardware, capital software, capital maintenance, recurrent training, consumables, personnel, and direct support.

Review the costing purpose and scope identified in Worksheet 1.1. You may skip sections for costs categories that were excluded from the scope of costing activities. Where services are provided by an external contractor, you may choose to skip questions that require contacting the contractor, if your costing purpose requires only the gross payments made to contractors and not a full enumeration of all line item costs.

Download the costing spreadsheet attached as a supplementary file with this guide. Throughout the guide, questions written in blue text ask about resources used in service provision. Record these resources as line items under each cost category tab in the spreadsheet. You may take notes directly in the spreadsheet, or record notes separately and transfer to the spreadsheet later. Review the spreadsheet before conducting the interview.

Participants should be identified from Worksheet 2.1: Facility informant identification. Each section suggests a participant, but additional or alternative participants may be needed to fully answer all questions. You may administer each section of the guide to participants individually, or assemble participants for all participants at once to complete the guide together in a focus group.

Where possible and with the consent of the interviewee, we recommend audio-recording the interviews so that recordings may be reviewed later for clarification or to extract additional detail, if necessary.

### Capital hardware

*The following section should be completed by an administrator, environmental health officer, or clinical supervisor with knowledge of water supply. For text in blue, record the responses as line items under the Capital Hardware tab of the costing spreadsheet.*

Name of Respondent:

Position/Title of Respondent:

Phone Number of Respondent:

Email of Respondent (if available):

Date of Interview:

1. What is the main water source for this facility? Describe the infrastructure.
2. How is water distributed in the facility? Describe the infrastructure.
3. How is water accessed at the point of care? Describe the infrastructure.
4. How is drinking water accessed by facility staff, patients, and caregivers? Describe the infrastructure.
5. How is water stored for emergencies? Describe the infrastructure.
6. How is water treated to be safe for drinking? For medical procedures? Describe the infrastructure.

### Capital software

*The following section should be completed by an administrator, environmental health officer, or clinical supervisor with knowledge of how water supply infrastructure was constructed or an individual who has access to relevant records of construction. For text in blue, record the responses as line items under the Capital Software tab of the costing spreadsheet.*

Name of Respondent:

Position/Title of Respondent:

Phone Number of Respondent:

Email of Respondent (if available):

Date of Interview:

1. Describe any site assessments, planning activities, or architectural design processes conducted before installing the water source.
2. Describe any site assessments, planning activities, or architectural design processes conducted before installing the water distribution pipes.
3. Describe any site assessments, planning activities, or architectural design processes conducted before installing the water access at the point of care.
4. Describe any site assessments, planning activities, or architectural design processes conducted before installing the water access for drinking.
5. Describe any site assessments, planning activities, or architectural design processes conducted before installing the emergency storage infrastructure.
6. Describe any site assessments, planning activities, or architectural design processes conducted before installing the water treatment infrastructure.

### Capital maintenance

*The following section to be completed with by an individual with knowledge of maintenance and repairs for the water system. For text in blue, record the responses as line items under the Capital Maintenance tab of the costing spreadsheet.*

Name of Respondent:

Position/Title of Respondent:

Phone Number of Respondent:

Email of Respondent (if available):

Date of Interview:

1. Is maintenance of the main water point performed in-house or by a contractor?

*If performed in-house:*

- 1. What, if any, regular maintenance (including cleaning) is performed on the main water point?
     1. What maintenance is performed daily?
     2. What maintenance is performed weekly?
     3. What maintenance is performed monthly?
     4. What maintenance is performed yearly?
     5. What major repairs, renovations, or rehabilitations are needed on the main water point over the course of its lifespan?

*If an external contractor is used:*

- 1. How are payments to the contractor made? What payments are made?
  2. What is the name of the contractor and what is their contact information?

**Note:** Add this information to the contractor and local partner identification worksheet if not already there. If detailed information on line items costs is needed, contact the contractor and ask questions 1.1.–1.2.

1. Is maintenance of the water distribution system performed in-house or by a contractor?

*If performed in-house:*

- 1. What, if any, regular maintenance (including cleaning) is performed on the water distribution system?
     1. What maintenance is performed daily?
     2. What maintenance is performed weekly?
     3. What maintenance is performed monthly?
     4. What maintenance is performed yearly?
     5. What major repairs, renovations, or rehabilitations are needed on the water distribution system over the course of its lifespan?

*If an external contractor is used:*

- 1. How are payments to the contractor made? What payments are made?
  2. What is the name of the contractor and what is their contact information?

**Note:** Add this information to the contractor and local partner identification worksheet if not already there. If detailed information on line items costs is needed, contact the contractor and ask questions 1.1.–1.2.

1. Is maintenance of the water access points at the point of care performed in-house or by a contractor?

*If performed in-house:*

- 1. What, if any, regular maintenance (including cleaning) is performed on the water access points at the point of care?
     1. What maintenance is performed daily?
     2. What maintenance is performed weekly?
     3. What maintenance is performed monthly?
     4. What maintenance is performed yearly?
     5. What major repairs, renovations, or rehabilitations are needed on the water access at the point of care over the course of their lifespan?

*If an external contractor is used:*

- 1. How are payments to the contractor made? What payments are made?
  2. What is the name of the contractor and what is their contact information?

**Note:** Add this information to the contractor and local partner identification worksheet if not already there. If detailed information on line items costs is needed, contact the contractor and ask questions 2.1–2.2.

1. Is maintenance of the drinking water access points performed in-house or by a contractor?

*If performed in-house:*

- 1. What, if any, regular maintenance (including cleaning) is performed on the drinking water access points?
     1. What maintenance is performed daily?
     2. What maintenance is performed weekly?
     3. What maintenance is performed monthly?
     4. What maintenance is performed yearly?
     5. What major repairs, renovations, or rehabilitations are needed on the drinking water access points over the course of their lifespan?

*If an external contractor is used:*

- 1. How are payments to the contractor made? What payments are made?
  2. What is the name of the contractor and what is their contact information?

**Note:** Add this information to the contractor and local partner identification worksheet if not already there. If detailed information on line items costs is needed, contact the contractor and ask questions 3.1–3.2.

1. Is maintenance of the emergency storage system performed in-house or by a contractor?

*If performed in-house:*

- 1. What, if any, regular maintenance (including cleaning) is performed on the emergency storage system?
     1. What maintenance is performed daily?
     2. What maintenance is performed weekly?
     3. What maintenance is performed monthly?
     4. What maintenance is performed yearly?
     5. What major repairs, renovations, or rehabilitations are needed on the emergency storage system over the course of its lifespan?

*If an external contractor is used:*

- 1. How are payments to the contractor made? What payments are made?
  2. What is the name of the contractor and what is their contact information?

**Note:** Add this information to the contractor and local partner identification worksheet if not already there. If detailed information on line items costs is needed, contact the contractor and ask questions 4.1–4.2.

1. Is maintenance of the water treatment system performed in-house or by a contractor?

*If performed in-house:*

- 1. What, if any, regular maintenance (including cleaning) is performed on the water treatment system?
     1. What maintenance is performed daily?
     2. What maintenance is performed weekly?
     3. What maintenance is performed monthly?
     4. What maintenance is performed yearly?
     5. What major repairs, renovations, or rehabilitations are needed on the water treatment system over the course of its lifespan?

*If an external contractor is used:*

- 1. How are payments to the contractor made? What payments are made?
  2. What is the name of the contractor and what is their contact information?

**Note:** Add this information to the contractor and local partner identification worksheet if not already there. If detailed information on line items costs is needed, contact the contractor and ask questions 4.1–4.2.

### Recurrent training

*The following section should be completed by an administrator, environmental health officer, or clinical supervisor who has knowledge of trainings. For text in blue, record the responses as line items under the Recurrent Training tab of the costing spreadsheet.*

Name of Respondent:

Position/Title of Respondent:

Phone Number of Respondent:

Email of Respondent (if available):

Date of Interview:

1. What routine training is conducted for water source safety, testing, and operation?
2. Are any other trainings conducted related to water? If so, what?

### Consumables

*The following section should be completed by an administrator, environmental health officer, or clinical supervisor with knowledge of water supply. For text in blue, record the responses as line items under the Consumables tab of the costing spreadsheet. Line items in this section should be consumable products (i.e., products and supplies that are consumed during normal operation; e.g., disposable cups). Tools, equipment, or supplies that are re-used without being consumed over the course of normal operation are considered capital hardware and should be logged under the capital hardware tab.*

Name of Respondent:

Position/Title of Respondent:

Phone Number of Respondent:

Email of Respondent (if available):

Date of Interview:

1. What water utilities are paid at this facility?
2. Who manages the main water point for this facility?

*If facility staff manage the main water point:*

- 1. How is water from the main water point treated?
     1. What products and supplies (including personal protective equipment) are used for water treatment?
  2. How is water from the main water point tested for quality?
     1. What products and supplies (including personal protective equipment) are used for water testing?

*If a contractor external to the facility, such as a government or privately owned utility or a community water committee manages the main water point:*

- 1. What payments are made to the contractor? Who makes the payments?
  2. What is the name of the system operator and what is their contact information?

**Note:** Add this information to the contractor and local partner identification worksheet if not already there. If detailed information on line items costs is needed, contact the contractor and ask questions 5.1–5.2.

1. Is water ever tested or treated again it is distributed in the facility?
   1. What products and supplies (including personal protective equipment) are used for water testing and/or treatment within the facility?
2. What products and supplies are used for water access at the point of care, including cleaning and restocking of any storage containers.
3. What products and supplies are used for drinking water access, include cups and utensils for drinking, supplies for cleaning and restocking containers.
4. What products and supplies are used for emergency storage containers, including cleaning and restocking containers?

### Personnel

*The following section should be completed by an administrator, environmental health officer, or clinical supervisor who supervises personnel. For text in blue, record the responses as line items under the Personnel tab of the costing spreadsheet. For all of the following questions, document the title/position of the person performing the task (e.g., laboratory technician, groundskeeper), not their specific name.*

Name of Respondent:

Position/Title of Respondent:

Phone Number of Respondent:

Email of Respondent (if available):

Date of Interview:

1. Who performs the following tasks:

**Note:** Document the title/position of the person performing the task (e.g., laboratory technician, groundskeeper), not their specific name.

- 1. Water testing and treatment?
  2. Collecting and transporting water to the facility?
  3. Cleaning and restocking of water containers at the point of care?
  4. Cleaning and restocking drinking water containers?
  5. Cleaning, flushing, and refilling of emergency storage containers?

1. Does anyone else perform water-related tasks? If so, what tasks? Who?

### Direct support

*The following section should be completed by an administrator, environmental health officer, or clinical supervisor who supervises water supply. For text in blue, record the responses as line items under the Direct Support tab of the costing spreadsheet. For all questions about people, document the title/position of the person performing the task (e.g., laboratory technician, groundskeeper), not their specific name.*

Name of Respondent:

Position/Title of Respondent:

Phone Number of Respondent:

Email of Respondent (if available):

Date of Interview:

1. Who supervises the following:
   1. Operation and functionality of the main water point?
   2. Water testing and treatment?
   3. Collection and transport of water to the facility?
   4. Stocking and availability of water at the point of care?
   5. Stocking and availability of drinking water containers?
   6. Stocking and availability of emergency storage containers?
2. Who is responsible for logistics and procurement of water-related supplies?
3. Does this facility have a water safe plan? If so, who develop and monitors the plan?
4. Does anyone else supervise water-related tasks? If so, what tasks? Who?
5. Are any tools, equipment, or supplies required for audits, safety monitoring, or supervision of water you have not already described? If so, please describe.

# Worksheet 5.2a: Sanitation line item identification (on site)

## Instructions

The purpose of this interview is to identify the resources used to provide sanitation. These resources will be entered as line items into a costing spreadsheet. Later modules will add information about quantities and costs of each item to calculate total costs.

Read this interview guide in its entirety before conducting the interview. The guide is divided into sections for the following cost categories: capital hardware, capital software, capital maintenance, recurrent training, consumables, personnel, and direct support.

Review the costing purpose and scope identified in Worksheet 1.1. You may skip sections for costs categories that were excluded from the scope of costing activities. Where services are provided by an external contractor, you may choose to skip questions that require contacting the contractor, if your costing purpose requires only the gross payments made to contractors and not a full enumeration of all line item costs.

Download the costing spreadsheet attached as a supplementary file with this guide. Throughout the guide, questions written in blue text ask about resources used in service provision. Record these resources as line items under each cost category tab in the spreadsheet. You may take notes directly in the spreadsheet, or record notes separately and transfer to the spreadsheet later. Review the spreadsheet before conducting the interview.

Participants should be identified from Worksheet 2.1: Facility informant identification. Each section suggests a participant, but additional or alternative participants may be needed to fully answer all questions. You may administer each section of the guide to participants individually, or assemble participants for all participants at once to complete the guide together in a focus group.

Where possible and with the consent of the interviewee, we recommend audio-recording the interviews so that recordings may be reviewed later for clarification or to extract additional detail, if necessary.

### Capital hardware

*The following section should be completed by an administrator, environmental health officer, or clinical supervisor with knowledge of sanitation. For text in blue, record the responses as line items under the Capital Hardware tab of the costing spreadsheet.*

Name of Respondent:

Position/Title of Respondent:

Phone Number of Respondent:

Email of Respondent (if available):

Date of Interview:

1. What toilets are present at this facility? Describe the infrastructure.
2. Do these toilets connect to a septic tank? If yes, describe the infrastructure.
3. What facilities for menstrual hygiene management are present at this facility? Describe the infrastructure.
4. What facilities are available near toilets for handwashing? Describe the infrastructure.

### Capital software

*The following section should be completed by an administrator, environmental health officer, or clinical supervisor with knowledge of how sanitation infrastructure was constructed or an individual who has access to relevant records of construction. For text in blue, record the responses as line items under the Capital Software tab of the costing spreadsheet.*

Name of Respondent:

Position/Title of Respondent:

Phone Number of Respondent:

Email of Respondent (if available):

Date of Interview:

1. Describe any site assessments, planning activities, or architectural design processes conducted before installing the toilets.
2. Describe any site assessments, planning activities, or architectural design processes conducted before installing the septic tanks.
3. Describe any site assessments, planning activities, or architectural design processes conducted before installing the menstrual hygiene management facilities.
4. Describe any site assessments, planning activities, or architectural design processes conducted before installing the handwashing facilities available near toilets.

### Capital maintenance

*The following section to be completed with by an individual with knowledge of maintenance and repairs for the sanitation facilities. For text in blue, record the responses as line items under the Capital Maintenance tab of the costing spreadsheet.*

Name of Respondent:

Position/Title of Respondent:

Phone Number of Respondent:

Email of Respondent (if available):

Date of Interview:

1. Is maintenance of the toilets performed in-house or by a contractor?

*If performed in-house:*

- 1. What, if any, regular maintenance (including cleaning) is performed on the toilets?
     1. What maintenance is performed daily?
     2. What maintenance is performed weekly?
     3. What maintenance is performed monthly?
     4. What maintenance is performed yearly?
     5. What major repairs, renovations, or rehabilitations are needed on the toilets over the course of their lifespan?

*If an external contractor is used:*

- 1. How are payments to the contractor made? What payments are made?
  2. What is the name of the contractor and what is their contact information?

**Note:** Add this information to the contractor and local partner identification worksheet if not already there. If detailed information on line items costs is needed, contact the contractor and ask questions 1.1.–1.2.

1. Is maintenance of the septic tank performed in-house or by a contractor?

*If performed in-house:*

- 1. What, if any, regular maintenance (including cleaning) is performed on the septic tank?
     1. What maintenance is performed daily?
     2. What maintenance is performed weekly?
     3. What maintenance is performed monthly?
     4. What maintenance is performed yearly?
     5. What major repairs, renovations, or rehabilitations are needed on the sanitation facilities over the course of their lifespan?

*If an external contractor is used:*

- 1. How are payments to the contractor made? What payments are made?
  2. What is the name of the contractor and what is their contact information?

**Note:** Add this information to the contractor and local partner identification worksheet if not already there. If detailed information on line items costs is needed, contact the contractor and ask questions 1.1.–1.2.

1. When toilet pits or the septic tank are filled, how are they emptied?
2. Is maintenance of the menstrual hygiene management facilities performed in-house or by a contractor?

*If performed in-house:*

- 1. What, if any, regular maintenance (including cleaning) is performed on the menstrual hygiene management facilities?
     1. What maintenance is performed daily?
     2. What maintenance is performed weekly?
     3. What maintenance is performed monthly?
     4. What maintenance is performed yearly?
     5. What major repairs, renovations, or rehabilitations are needed on the menstrual hygiene management facilities over the course of their lifespan?

*If an external contractor is used:*

- 1. How are payments to the contractor made? What payments are made?
  2. What is the name of the contractor and what is their contact information?

**Note:** Add this information to the contractor and local partner identification worksheet if not already there. If detailed information on line items costs is needed, contact the contractor and ask questions 1.1.–1.2.

1. Is maintenance of the handwashing facilities near toilets performed in-house or by a contractor?

*If performed in-house:*

- 1. What, if any, regular maintenance (including cleaning) is performed on the handwashing facilities?
     1. What maintenance is performed daily?
     2. What maintenance is performed weekly?
     3. What maintenance is performed monthly?
     4. What maintenance is performed yearly?
     5. What major repairs, renovations, or rehabilitations are needed on the handwashing facilities over the course of their lifespan?

*If an external contractor is used:*

- 1. How are payments to the contractor made? What payments are made?
  2. What is the name of the contractor and what is their contact information?

**Note:** Add this information to the contractor and local partner identification worksheet if not already there. If detailed information on line items costs is needed, contact the contractor and ask questions 1.1.–1.2.

### Recurrent training

*The following section should be completed by an administrator, environmental health officer, or clinical supervisor who has knowledge of trainings. For text in blue, record the responses as line items under the Recurrent Training tab of the costing spreadsheet.*

Name of Respondent:

Position/Title of Respondent:

Phone Number of Respondent:

Email of Respondent (if available):

Date of Interview:

1. Are any trainings conducted related to sanitation? If so, what?
2. What materials are present at sanitation facilities to promote handwashing?

### Consumables

*The following section should be completed by an administrator, environmental health officer, or clinical supervisor with knowledge of sanitation facilities. For text in blue, record the responses as line items under the Consumables tab of the costing spreadsheet. Line items in this section should be consumable products (i.e., products and supplies that are consumed during normal operation; e.g., anal cleansing materials). Tools, equipment, or supplies that are re-used without being consumed over the course of normal operation are considered capital hardware and should be logged under the capital hardware tab.*

Name of Respondent:

Position/Title of Respondent:

Phone Number of Respondent:

Email of Respondent (if available):

Date of Interview:

1. What products and supplies are used for anal cleansing?
2. What products and supplies are provided for cleaning during menstrual hygiene management (e.g., soap, drying materials)?
3. What products (e.g., sanitary pads) are provided for menstrual hygiene management?
4. What utilities are paid for sanitation and menstrual hygiene management facilities? Consider water needed for flushing or cleaning, sewer utilities, electricity for lighting facilities.
5. What other products and supplies are needed for sanitation?
6. What products and supplies are available for handwashing and drying after defecation?

### Personnel

*The following section should be completed by an administrator, environmental health officer, or clinical supervisor who supervises personnel. For text in blue, record the responses as line items under the Personnel tab of the costing spreadsheet. For all of the following questions, document the title/position of the person performing the task (e.g., laboratory technician, groundskeeper), not their specific name.*

Name of Respondent:

Position/Title of Respondent:

Phone Number of Respondent:

Email of Respondent (if available):

Date of Interview:

1. Who performs the following tasks:

**Note:** Document the title/position of the person performing the task (e.g., laboratory technician, groundskeeper), not their specific name.

- 1. Restocking anal cleansing materials?
  2. Restocking soap and drying materials in menstrual hygiene management facilities?
  3. Emptying and disposal of receptacles for soiled menstrual products?
  4. Restocking hand washing and drying materials?
  5. Emptying and disposal of receptacles for soiled hand drying materials?

1. Does anyone else perform sanitation-related tasks? If so, what tasks? Who?

### Direct support

*The following section should be completed by an administrator, environmental health officer, or clinical supervisor who supervises sanitation. For text in blue, record the responses as line items under the Direct Support tab of the costing spreadsheet. For all questions about people, document the title/position of the person performing the task (e.g., laboratory technician, groundskeeper), not their specific name.*

Name of Respondent:

Position/Title of Respondent:

Phone Number of Respondent:

Email of Respondent (if available):

Date of Interview:

1. Who supervises the following:
   1. Sanitary inspections?
   2. Monitoring and inspections of hand hygiene compliance?
2. Does anyone else supervise sanitation-related tasks? If so, what tasks? Who?
3. Are any tools, equipment, or supplies required for audits, safety monitoring, or supervision of sanitation you have not already described? If so, please describe.

# Worksheet 5.2b: Sanitation line item identification (sewered)

## Instructions

The purpose of this interview is to identify the resources used to provide sanitation. These resources will be entered as line items into a costing spreadsheet. Later modules will add information about quantities and costs of each item to calculate total costs.

Read this interview guide in its entirety before conducting the interview. The guide is divided into sections for the following cost categories: capital hardware, capital software, capital maintenance, recurrent training, consumables, personnel, and direct support.

Review the costing purpose and scope identified in Worksheet 1.1. You may skip sections for costs categories that were excluded from the scope of costing activities. Where services are provided by an external contractor, you may choose to skip questions that require contacting the contractor, if your costing purpose requires only the gross payments made to contractors and not a full enumeration of all line item costs.

Download the costing spreadsheet attached as a supplementary file with this guide. Throughout the guide, questions written in blue text ask about resources used in service provision. Record these resources as line items under each cost category tab in the spreadsheet. You may take notes directly in the spreadsheet, or record notes separately and transfer to the spreadsheet later. Review the spreadsheet before conducting the interview.

Participants should be identified from Worksheet 2.1: Facility informant identification. Each section suggests a participant, but additional or alternative participants may be needed to fully answer all questions. You may administer each section of the guide to participants individually, or assemble participants for all participants at once to complete the guide together in a focus group.

Where possible and with the consent of the interviewee, we recommend audio-recording the interviews so that recordings may be reviewed later for clarification or to extract additional detail, if necessary.

### Capital hardware

*The following section should be completed by an administrator, environmental health officer, or clinical supervisor with knowledge of sanitation. For text in blue, record the responses as line items under the Capital Hardware tab of the costing spreadsheet.*

Name of Respondent:

Position/Title of Respondent:

Phone Number of Respondent:

Email of Respondent (if available):

Date of Interview:

1. What toilets are present at this facility? Describe the infrastructure.
2. How is sewage removed from the facility? Describe the infrastructure.
3. What facilities for menstrual hygiene management are present at this facility? Describe the infrastructure.
4. What facilities are available near toilets for handwashing? Describe the infrastructure.

### Capital software

*The following section should be completed by an administrator, environmental health officer, or clinical supervisor with knowledge of how sanitation infrastructure was constructed or an individual who has access to relevant records of construction. For text in blue, record the responses as line items under the Capital Software tab of the costing spreadsheet.*

Name of Respondent:

Position/Title of Respondent:

Phone Number of Respondent:

Email of Respondent (if available):

Date of Interview:

1. Describe any site assessments, planning activities, or architectural design processes conducted before installing the toilets.
2. Describe any site assessments, planning activities, or architectural design processes conducted before installing sewer pipes.
3. Describe any site assessments, planning activities, or architectural design processes conducted before installing the menstrual hygiene management facilities.
4. Describe any site assessments, planning activities, or architectural design processes conducted before installing the handwashing facilities available near toilets.

### Capital maintenance

*The following section to be completed with by an individual with knowledge of maintenance and repairs for the sanitation facilities. For text in blue, record the responses as line items under the Capital Maintenance tab of the costing spreadsheet.*

Name of Respondent:

Position/Title of Respondent:

Phone Number of Respondent:

Email of Respondent (if available):

Date of Interview:

1. Is maintenance of the toilets performed in-house or by a contractor?

*If performed in-house:*

- 1. What, if any, regular maintenance (including cleaning) is performed on the toilets?
     1. What maintenance is performed daily?
     2. What maintenance is performed weekly?
     3. What maintenance is performed monthly?
     4. What maintenance is performed yearly?
     5. What major repairs, renovations, or rehabilitations are needed on the toilets over the course of their lifespan?

*If an external contractor is used:*

- 1. How are payments to the contractor made? What payments are made?
  2. What is the name of the contractor and what is their contact information?

**Note:** Add this information to the contractor and local partner identification worksheet if not already there. If detailed information on line items costs is needed, contact the contractor and ask questions 1.1.–1.2.

1. Is maintenance of the sewer system performed in-house or by a contractor?

*If performed in-house:*

- 1. What, if any, regular maintenance (including cleaning) is performed on the sewer system?
     1. What maintenance is performed daily?
     2. What maintenance is performed weekly?
     3. What maintenance is performed monthly?
     4. What maintenance is performed yearly?
     5. What major repairs, renovations, or rehabilitations are needed on the sanitation facilities over the course of their lifespan?

*If an external contractor is used:*

- 1. How are payments to the contractor made? What payments are made?
  2. What is the name of the contractor and what is their contact information?

**Note:** Add this information to the contractor and local partner identification worksheet if not already there. If detailed information on line items costs is needed, contact the contractor and ask questions 1.1.–1.2.

1. Is maintenance of the menstrual hygiene management facilities performed in-house or by a contractor?

*If performed in-house:*

- 1. What, if any, regular maintenance (including cleaning) is performed on the menstrual hygiene management facilities?
     1. What maintenance is performed daily?
     2. What maintenance is performed weekly?
     3. What maintenance is performed monthly?
     4. What maintenance is performed yearly?
     5. What major repairs, renovations, or rehabilitations are needed on the menstrual hygiene management facilities over the course of their lifespan?

*If an external contractor is used:*

- 1. How are payments to the contractor made? What payments are made?
  2. What is the name of the contractor and what is their contact information?

**Note:** Add this information to the contractor and local partner identification worksheet if not already there. If detailed information on line items costs is needed, contact the contractor and ask questions 1.1.–1.2.

1. Is maintenance of the handwashing facilities near toilets performed in-house or by a contractor?

*If performed in-house:*

- 1. What, if any, regular maintenance (including cleaning) is performed on the handwashing facilities?
     1. What maintenance is performed daily?
     2. What maintenance is performed weekly?
     3. What maintenance is performed monthly?
     4. What maintenance is performed yearly?
     5. What major repairs, renovations, or rehabilitations are needed on the handwashing facilities over the course of their lifespan?

*If an external contractor is used:*

- 1. How are payments to the contractor made? What payments are made?
  2. What is the name of the contractor and what is their contact information?

**Note:** Add this information to the contractor and local partner identification worksheet if not already there. If detailed information on line items costs is needed, contact the contractor and ask questions 1.1.–1.2.

### Recurrent training

*The following section should be completed by an administrator, environmental health officer, or clinical supervisor who has knowledge of trainings. For text in blue, record the responses as line items under the Recurrent Training tab of the costing spreadsheet.*

Name of Respondent:

Position/Title of Respondent:

Phone Number of Respondent:

Email of Respondent (if available):

Date of Interview:

1. Are any trainings conducted related to sanitation? If so, what?
2. What materials are present at sanitation facilities to promote handwashing?

### Consumables

*The following section should be completed by an administrator, environmental health officer, or clinical supervisor with knowledge of sanitation facilities. For text in blue, record the responses as line items under the Consumables tab of the costing spreadsheet. Line items in this section should be consumable products (i.e., products and supplies that are consumed during normal operation; e.g., anal cleansing materials). Tools, equipment, or supplies that are re-used without being consumed over the course of normal operation are considered capital hardware and should be logged under the capital hardware tab.*

Name of Respondent:

Position/Title of Respondent:

Phone Number of Respondent:

Email of Respondent (if available):

Date of Interview:

1. What products and supplies are used for anal cleansing?
2. What products and supplies are provided for cleaning during menstrual hygiene management (e.g., soap, drying materials)?
3. What products (e.g., sanitary pads) are provided for menstrual hygiene management?
4. What utilities are paid for sanitation and menstrual hygiene management facilities? Consider water needed for flushing or cleaning, sewer utilities, electricity for lighting facilities.
5. What other products and supplies are needed for sanitation?
6. What products and supplies are available for handwashing and drying after defecation?

### Personnel

*The following section should be completed by an administrator, environmental health officer, or clinical supervisor who supervises personnel. For text in blue, record the responses as line items under the Personnel tab of the costing spreadsheet. For all of the following questions, document the title/position of the person performing the task (e.g., laboratory technician, groundskeeper), not their specific name.*

Name of Respondent:

Position/Title of Respondent:

Phone Number of Respondent:

Email of Respondent (if available):

Date of Interview:

1. Who performs the following tasks:

**Note:** Document the title/position of the person performing the task (e.g., laboratory technician, groundskeeper), not their specific name.

- 1. Restocking anal cleansing materials?
  2. Restocking soap and drying materials in menstrual hygiene management facilities?
  3. Emptying and disposal of receptacles for soiled menstrual products?
  4. Restocking hand washing and drying materials?
  5. Emptying and disposal of receptacles for soiled hand drying materials?

1. Does anyone else perform sanitation-related tasks? If so, what tasks? Who?

### Direct support

*The following section should be completed by an administrator, environmental health officer, or clinical supervisor who supervises sanitation. For text in blue, record the responses as line items under the Direct Support tab of the costing spreadsheet. For all questions about people, document the title/position of the person performing the task (e.g., laboratory technician, groundskeeper), not their specific name.*

Name of Respondent:

Position/Title of Respondent:

Phone Number of Respondent:

Email of Respondent (if available):

Date of Interview:

1. Who supervises the following:
   1. Sanitary inspections?
   2. Monitoring and inspections of hand hygiene compliance at sanitation facilities?
2. Does anyone else supervise sanitation-related tasks? If so, what tasks? Who?
3. Are any tools, equipment, or supplies required for audits, safety monitoring, or supervision of sanitation you have not already described? If so, please describe.

# Worksheet 5.3: Hygiene at points of care line item identification

## Instructions

The purpose of this interview is to identify the resources used to provide hygiene at points of care. These resources will be entered as line items into a costing spreadsheet. Later modules will add information about quantities and costs of each item to calculate total costs.

Read this interview guide in its entirety before conducting the interview. The guide is divided into sections for the following cost categories: capital hardware, capital software, capital maintenance, recurrent training, consumables, personnel, and direct support.

Review the costing purpose and scope identified in Worksheet 1.1. You may skip sections for costs categories that were excluded from the scope of costing activities. Where services are provided by an external contractor, you may choose to skip questions that require contacting the contractor, if your costing purpose requires only the gross payments made to contractors and not a full enumeration of all line item costs.

Download the costing spreadsheet attached as a supplementary file with this guide. Throughout the guide, questions written in blue text ask about resources used in service provision. Record these resources as line items under each cost category tab in the spreadsheet. You may take notes directly in the spreadsheet, or record notes separately and transfer to the spreadsheet later. Review the spreadsheet before conducting the interview.

Participants should be identified from Worksheet 2.1: Facility informant identification. Each section suggests a participant, but additional or alternative participants may be needed to fully answer all questions. You may administer each section of the guide to participants individually, or assemble participants for all participants at once to complete the guide together in a focus group.

Where possible and with the consent of the interviewee, we recommend audio-recording the interviews so that recordings may be reviewed later for clarification or to extract additional detail, if necessary.

### Capital hardware

*The following section should be completed by an administrator, environmental health officer, or clinical supervisor with knowledge of hygiene at points of care. For text in blue, record the responses as line items under the Capital Hardware tab of the costing spreadsheet.*

Name of Respondent:

Position/Title of Respondent:

Phone Number of Respondent:

Email of Respondent (if available):

Date of Interview:

1. How do healthcare workers wash their hands at the point of care? Describe the infrastructure.
2. How do healthcare workers access alcohol-based hand rub? Describe the infrastructure.
3. What reusable personal protective equipment is used by healthcare workers?

### Capital software

*The following section should be completed by an administrator, environmental health officer, or clinical supervisor with knowledge of how hygiene infrastructure was constructed or an individual who has access to relevant records of construction. For text in blue, record the responses as line items under the Capital Software tab of the costing spreadsheet.*

Name of Respondent:

Position/Title of Respondent:

Phone Number of Respondent:

Email of Respondent (if available):

Date of Interview:

1. Describe any site assessments, planning activities, or architectural design processes conducted before installing handwashing stations at points of care.
2. Describe any site assessments, planning activities, or architectural design processes conducted before installing alcohol-based hand rub dispensers.
3. Describe any orientation needed to train staff on alcohol-based hand rub formulation and stocking.

### Capital maintenance

*The following section to be completed with by an individual with knowledge of maintenance and repairs for the hygiene facilities. For text in blue, record the responses as line items under the Capital Maintenance tab of the costing spreadsheet.*

Name of Respondent:

Position/Title of Respondent:

Phone Number of Respondent:

Email of Respondent (if available):

Date of Interview:

1. Is maintenance of handwashing stations at points of care performed in-house or by a contractor?

*If performed in-house:*

- 1. What, if any, regular maintenance (including cleaning) is performed on handwashing stations at points of care?
     1. What maintenance is performed daily?
     2. What maintenance is performed weekly?
     3. What maintenance is performed monthly?
     4. What maintenance is performed yearly?
     5. What major repairs, renovations, or rehabilitations are needed on handwashing stations at points of care over the course of their lifespan?

*If an external contractor is used:*

- 1. How are payments to the contractor made? What payments are made?
  2. What is the name of the contractor and what is their contact information?

**Note:** Add this information to the contractor and local partner identification worksheet if not already there. If detailed information on line items costs is needed, contact the contractor and ask questions 1.1.–1.2.

1. Is maintenance of alcohol hand rub dispensers performed in-house or by a contractor?

*If performed in-house:*

- 1. What, if any, regular maintenance (including cleaning) is performed on alcohol hand rub dispensers?
     1. What maintenance is performed daily?
     2. What maintenance is performed weekly?
     3. What maintenance is performed monthly?
     4. What maintenance is performed yearly?
     5. What major repairs, renovations, or rehabilitations are needed on alcohol hand rub dispensers over the course of their lifespan?

*If an external contractor is used:*

- 1. How are payments to the contractor made? What payments are made?
  2. What is the name of the contractor and what is their contact information?

**Note:** Add this information to the contractor and local partner identification worksheet if not already there. If detailed information on line items costs is needed, contact the contractor and ask questions 1.1.–1.2.

1. What is the process for decontaminating, sterilizing, and restocking soiled reusable PPE?
2. Are reusable personal protective equipment repaired when damaged? If so, what repairs are done?

### Recurrent training

*The following section should be completed by an administrator, environmental health officer, or clinical supervisor who with knowledge of trainings. For text in blue, record the responses as line items under the Recurrent Training tab of the costing spreadsheet.*

Name of Respondent:

Position/Title of Respondent:

Phone Number of Respondent:

Email of Respondent (if available):

Date of Interview:

1. What routine training is conducted for proper handwashing technique and handwashing promotion?
2. What routine training is conducted for proper personal protective equipment use?

### Consumables

*The following section should be completed by an administrator, environmental health officer, or clinical supervisor with knowledge of hygiene facilities. For text in blue, record the responses as line items under the Consumables tab of the costing spreadsheet. Line items in this section should be consumable products (i.e., products and supplies that are consumed during normal operation; e.g., anal cleansing materials). Tools, equipment, or supplies that are re-used without being consumed over the course of normal operation are considered capital hardware and should be logged under the capital hardware tab.*

Name of Respondent:

Position/Title of Respondent:

Phone Number of Respondent:

Email of Respondent (if available):

Date of Interview:

1. What products and supplies are used for handwashing at points of care (e.g., soap, drying materials)?
2. What products and supplies are used for alcohol-based hand rub?
3. What disposable personal protective equipment are used by healthcare workers during care provision?

### Personnel

*The following section should be completed by an administrator, environmental health officer, or clinical supervisor who supervises personnel. For text in blue, record the responses as line items under the Personnel tab of the costing spreadsheet. For all of the following questions, document the title/position of the person performing the task (e.g., laboratory technician, groundskeeper), not their specific name.*

Name of Respondent:

Position/Title of Respondent:

Phone Number of Respondent:

Email of Respondent (if available):

Date of Interview:

1. Who performs the following tasks:

**Note:** Document the title/position of the person performing the task (e.g., laboratory technician, groundskeeper), not their specific name.

- 1. Restocking hand washing and drying materials at points of care?
  2. Emptying and disposal of receptacles for soiled hand drying materials?
  3. Restocking alcohol-based hand rub?
  4. Restocking disposable personal protective equipment?

1. Does anyone else perform sanitation-related tasks? If so, what tasks? Who?

### Direct support

*The following section should be completed by an administrator, environmental health officer, or clinical supervisor who supervises sanitation. For text in blue, record the responses as line items under the Direct Support tab of the costing spreadsheet. For all questions about people, document the title/position of the person performing the task (e.g., laboratory technician, groundskeeper), not their specific name.*

Name of Respondent:

Position/Title of Respondent:

Phone Number of Respondent:

Email of Respondent (if available):

Date of Interview:

1. Who supervises the following:
   1. Monitoring and inspections of hand hygiene compliance?
   2. Monitoring and inspections of PPE compliance?
   3. Infection prevention and control planning?
2. Does anyone else supervise hygiene-related tasks? If so, what tasks? Who?
3. Are any tools, equipment, or supplies required for audits, safety monitoring, or supervision of hygiene and PPE use you have not already described? If so, please describe.

# Worksheet 5.4: Waste management line item identification

## Instructions

The purpose of this interview is to identify the resources used for waste management. These resources will be entered as line items into a costing spreadsheet. Later modules will add information about quantities and costs of each item to calculate total costs.

Read this interview guide in its entirety before conducting the interview. The guide is divided into sections for the following cost categories: capital hardware, capital software, capital maintenance, recurrent training, consumables, personnel, and direct support.

Review the costing purpose and scope identified in Worksheet 1.1. You may skip sections for costs categories that were excluded from the scope of costing activities. Where services are provided by an external contractor, you may choose to skip questions that require contacting the contractor, if your costing purpose requires only the gross payments made to contractors and not a full enumeration of all line item costs.

Download the costing spreadsheet attached as a supplementary file with this guide. Throughout the guide, questions written in blue text ask about resources used in service provision. Record these resources as line items under each cost category tab in the spreadsheet. You may take notes directly in the spreadsheet, or record notes separately and transfer to the spreadsheet later. Review the spreadsheet before conducting the interview.

Participants should be identified from Worksheet 2.1: Facility informant identification. Each section suggests a participant, but additional or alternative participants may be needed to fully answer all questions. You may administer each section of the guide to participants individually, or assemble participants for all participants at once to complete the guide together in a focus group.

Where possible and with the consent of the interviewee, we recommend audio-recording the interviews so that recordings may be reviewed later for clarification or to extract additional detail, if necessary.

Note that different types of waste (e.g., general, sharps, infectious) may be processed in different ways. Repeat the questions in this worksheet as necessary to determine resources used for all waste management processes.

### Capital hardware

*The following section should be completed by an administrator, environmental health officer, or clinical supervisor with knowledge of waste management. For text in blue, record the responses as line items under the Capital Hardware tab of the costing spreadsheet.*

Name of Respondent:

Position/Title of Respondent:

Phone Number of Respondent:

Email of Respondent (if available):

Date of Interview:

1. How is waste segregated at the point of care? Describe the infrastructure and equipment.
2. How is waste collected at the point of care and transported to its next destination? Describe the infrastructure and equipment.
3. Is waste stored at this facility prior to treatment? If so, describe the infrastructure and equipment.
4. If waste is stored prior to treatment, how is it transported from the storage site to the treatment site? Describe the infrastructure and equipment.
5. How is waste treated prior to disposal? Describe the infrastructure and equipment. If multiple treatment methods are used, describe all.
6. Once waste is treated, how is it transported to the final disposal site? Describe the infrastructure and equipment.
7. How is waste disposed after treatment? Describe the infrastructure and equipment.
8. What reusable personal protective equipment are used by staff when performing waste management duties?

### Capital software

*The following section should be completed by an administrator, environmental health officer, or clinical supervisor with knowledge of how waste management infrastructure was constructed or an individual who has access to relevant records of construction. For text in blue, record the responses as line items under the Capital Software tab of the costing spreadsheet.*

Name of Respondent:

Position/Title of Respondent:

Phone Number of Respondent:

Email of Respondent (if available):

Date of Interview:

1. Describe any orientations needed when waste segregation, treatment, and transportation equipment was installed.
2. Describe any site assessments, planning activities, or architectural design processes conducted before constructing the waste storage area.
3. Describe any site assessments, planning activities, or architectural design processes conducted before installing waste treatment equipment.
4. Describe any site assessments, planning activities, or architectural design processes conducted before constructing final disposal sites for waste.
5. Describe any licensing and registration fees needed for waste transportation vehicles.

### Capital maintenance

*The following section to be completed with by an individual with knowledge of maintenance and repairs for the waste management infrastructure and equipment. For text in blue, record the responses as line items under the Capital Maintenance tab of the costing spreadsheet.*

Name of Respondent:

Position/Title of Respondent:

Phone Number of Respondent:

Email of Respondent (if available):

Date of Interview:

1. Is maintenance of waste collection and segregation infrastructure and equipment performed in-house or by a contractor?

*If performed in-house:*

- 1. What, if any, regular maintenance (including cleaning) is performed on waste collection and segregation infrastructure and equipment?
     1. What maintenance is performed daily?
     2. What maintenance is performed weekly?
     3. What maintenance is performed monthly?
     4. What maintenance is performed yearly?
     5. What major repairs, renovations, or rehabilitations are needed on waste collection and segregation infrastructure and equipment over the course of their lifespan?

*If an external contractor is used:*

- 1. How are payments to the contractor made? What payments are made?
  2. What is the name of the contractor and what is their contact information?

**Note:** Add this information to the contractor and local partner identification worksheet if not already there. If detailed information on line items costs is needed, contact the contractor and ask questions 1.1.–1.2.

1. Is maintenance of waste transportation infrastructure and equipment performed in-house or by a contractor? Consider transportation equipment for transporting waste within facilities (e.g., carts and trolleys) and between facilities (e.g., trucks and trailers)

*If performed in-house:*

- 1. What, if any, regular maintenance (including cleaning) is performed on waste transportation infrastructure and equipment?
     1. What maintenance is performed daily?
     2. What maintenance is performed weekly?
     3. What maintenance is performed monthly?
     4. What maintenance is performed yearly?
     5. What major repairs, renovations, or rehabilitations are needed on waste transportation infrastructure and equipment over the course of their lifespan?

*If an external contractor is used:*

- 1. How are payments to the contractor made? What payments are made?
  2. What is the name of the contractor and what is their contact information?

**Note:** Add this information to the contractor and local partner identification worksheet if not already there. If detailed information on line items costs is needed, contact the contractor and ask questions 1.1.–1.2.

1. Is maintenance of waste treatment infrastructure and equipment performed in-house or by a contractor?

*If performed in-house:*

- 1. What, if any, regular maintenance (including cleaning) is performed on waste treatment infrastructure and equipment?
     1. What maintenance is performed daily?
     2. What maintenance is performed weekly?
     3. What maintenance is performed monthly?
     4. What maintenance is performed yearly?
     5. What major repairs, renovations, or rehabilitations are needed on waste treatment infrastructure and equipment over the course of their lifespan?

*If an external contractor is used:*

- 1. How are payments to the contractor made? What payments are made?
  2. What is the name of the contractor and what is their contact information?

**Note:** Add this information to the contractor and local partner identification worksheet if not already there. If detailed information on line items costs is needed, contact the contractor and ask questions 1.1.–1.2.

1. Is maintenance of waste final disposal infrastructure and equipment performed in-house or by a contractor?

*If performed in-house:*

- 1. What, if any, regular maintenance (including cleaning) is performed on waste final disposal infrastructure and equipment?
     1. What maintenance is performed daily?
     2. What maintenance is performed weekly?
     3. What maintenance is performed monthly?
     4. What maintenance is performed yearly?
     5. What major repairs, renovations, or rehabilitations are needed on waste final disposal infrastructure and equipment over the course of their lifespan?

*If an external contractor is used:*

- 1. How are payments to the contractor made? What payments are made?
  2. What is the name of the contractor and what is their contact information?

**Note:** Add this information to the contractor and local partner identification worksheet if not already there. If detailed information on line items costs is needed, contact the contractor and ask questions 1.1.–1.2.

### Recurrent training

*The following section should be completed by an administrator, environmental health officer, or clinical supervisor who with knowledge of trainings. For text in blue, record the responses as line items under the Recurrent Training tab of the costing spreadsheet.*

Name of Respondent:

Position/Title of Respondent:

Phone Number of Respondent:

Email of Respondent (if available):

Date of Interview:

1. What routine training is conducted for proper waste handling? Consider trainings for healthcare workers generating and segregating waste, waste transportation staff, waste treatment staff, and staff managing final disposal.
2. Are any other routine trainings conducted related to waste management?

### Consumables

*The following section should be completed by an administrator, environmental health officer, or clinical supervisor with knowledge of waste management. For text in blue, record the responses as line items under the Consumables tab of the costing spreadsheet. Line items in this section should be consumable products (i.e., products and supplies that are consumed during normal operation; e.g., anal cleansing materials). Tools, equipment, or supplies that are re-used without being consumed over the course of normal operation are considered capital hardware and should be logged under the capital hardware tab.*

Name of Respondent:

Position/Title of Respondent:

Phone Number of Respondent:

Email of Respondent (if available):

Date of Interview:

1. What products and supplies are used for collecting and segregating waste at the point of care?
2. What products and supplies are used for packing, labeling, and transporting waste within the facility?
3. What products and supplies are used for packing, labeling, and transporting waste between facilities?
4. What utilities are paid for operation of waste storage, treatment, and disposal equipment? Consider water, electric utilities, fuel needs, and other utility costs.
5. What disposable personal protective equipment are used by staff when performing waste management duties?

### Personnel

*The following section should be completed by an administrator, environmental health officer, or clinical supervisor who supervises personnel. For text in blue, record the responses as line items under the Personnel tab of the costing spreadsheet. For all of the following questions, document the title/position of the person performing the task (e.g., laboratory technician, groundskeeper), not their specific name.*

Name of Respondent:

Position/Title of Respondent:

Phone Number of Respondent:

Email of Respondent (if available):

Date of Interview:

1. Who performs the following tasks:

**Note:** Document the title/position of the person performing the task (e.g., laboratory technician, groundskeeper), not their specific name.

- 1. Collecting and packing waste from the point of care?
  2. Collecting and packing waste from storage areas?
  3. Transporting waste?
  4. Treating waste?
  5. Final disposal of waste?

1. Does anyone else perform waste management-related tasks? If so, what tasks? Who?

### Direct support

*The following section should be completed by an administrator, environmental health officer, or clinical supervisor who supervises waste management. For text in blue, record the responses as line items under the Direct Support tab of the costing spreadsheet. For all questions about people, document the title/position of the person performing the task (e.g., laboratory technician, groundskeeper), not their specific name.*

Name of Respondent:

Position/Title of Respondent:

Phone Number of Respondent:

Email of Respondent (if available):

Date of Interview:

1. Who supervises the following:
   1. Safety monitoring and audits of waste management?
2. Does anyone else supervise cleaning-related tasks? If so, what tasks? Who?
3. What recurring inspection, licensing, insurance, or other fees are needed for waste management equipment (e.g., waste transportation vehicle inspections, incinerator emissions testing)?
4. Do staff involved in waste management receive immunizations? If so, which immunizations?
5. Are any other tools, equipment, or supplies required for audits, safety monitoring, or supervision of waste management that you have not already described? If so, please describe.

# Worksheet 5.5: Environmental cleaning line item identification

## Instructions

The purpose of this interview is to identify the resources used for cleaning. These resources will be entered as line items into a costing spreadsheet. Later modules will add information about quantities and costs of each item to calculate total costs.

Read this interview guide in its entirety before conducting the interview. The guide is divided into sections for the following cost categories: capital hardware, capital software, capital maintenance, recurrent training, consumables, personnel, and direct support.

Review the costing purpose and scope identified in Worksheet 1.1. You may skip sections for costs categories that were excluded from the scope of costing activities. Where services are provided by an external contractor, you may choose to skip questions that require contacting the contractor, if your costing purpose requires only the gross payments made to contractors and not a full enumeration of all line item costs.

Download the costing spreadsheet attached as a supplementary file with this guide. Throughout the guide, questions written in blue text ask about resources used in service provision. Record these resources as line items under each cost category tab in the spreadsheet. You may take notes directly in the spreadsheet, or record notes separately and transfer to the spreadsheet later. Review the spreadsheet before conducting the interview.

Participants should be identified from Worksheet 2.1: Facility informant identification. Each section suggests a participant, but additional or alternative participants may be needed to fully answer all questions. You may administer each section of the guide to participants individually, or assemble participants for all participants at once to complete the guide together in a focus group.

Where possible and with the consent of the interviewee, we recommend audio-recording the interviews so that recordings may be reviewed later for clarification or to extract additional detail, if necessary.

Note that different types of waste (e.g., general, sharps, infectious) may be processed in different ways. Repeat the questions in this worksheet as necessary to determine resources used for all waste management processes.

### Capital hardware

*The following section should be completed by an administrator, environmental health officer, or clinical supervisor with knowledge of cleaning. For text in blue, record the responses as line items under the Capital Hardware tab of the costing spreadsheet.*

Name of Respondent:

Position/Title of Respondent:

Phone Number of Respondent:

Email of Respondent (if available):

Date of Interview:

1. Briefly describe the process for general cleaning (low level, high level, floors, walls, doors)? Describe the tools and equipment used.
2. Briefly describe the process for cleaning infectious waste and blood spills? Describe the tools and equipment used.
3. Briefly describe the process for cleaning surfaces with patient contact (e.g., mattresses, delivery beds)? Describe the tools and equipment used.
4. Does this facility have a sluice room or other designated area for disinfection and sterilization of equipment and devices? A sluice room is an area used for disposal of waste products and disinfection of associated equipment, also called a dirty utility room. Describe infrastructure and equipment in this area.
5. Briefly describe the process for disinfecting and sterilizing medical devices and equipment? Describe the tools and equipment used.
6. Briefly describe the process for cleaning toilets and handwash basins? Describe the tools and equipment used.
7. What reusable personal protective equipment are used by staff when performing cleaning duties?

### Capital software

*The following section should be completed by an administrator, environmental health officer, or clinical supervisor with knowledge of how sanitation infrastructure was constructed or an individual who has access to relevant records of construction. For text in blue, record the responses as line items under the Capital Software tab of the costing spreadsheet.*

Name of Respondent:

Position/Title of Respondent:

Phone Number of Respondent:

Email of Respondent (if available):

Date of Interview:

1. Describe any orientations to cleaning procedures and cleaning-related safety conducted for newly hired staff.
2. Describe any site assessments, planning activities, or architectural design processes conducted before constructing the sluice room or other designated disinfection and sterilization areas.

### Capital maintenance

*The following section to be completed with by an individual with knowledge of maintenance and repairs for the sanitation facilities. For text in blue, record the responses as line items under the Capital Maintenance tab of the costing spreadsheet.*

Name of Respondent:

Position/Title of Respondent:

Phone Number of Respondent:

Email of Respondent (if available):

Date of Interview:

1. Is maintenance of sluice room or other designated disinfection and sterilization area in-house or by a contractor?

*If performed in-house:*

- 1. What, if any, regular maintenance (including cleaning) is performed on infrastructure and equipment in this area?
     1. What maintenance is performed daily?
     2. What maintenance is performed weekly?
     3. What maintenance is performed monthly?
     4. What maintenance is performed yearly?
     5. What major repairs, renovations, or rehabilitations are needed on infrastructure and equipment in this area over the course of their lifespan?

*If an external contractor is used:*

- 1. How are payments to the contractor made? What payments are made?
  2. What is the name of the contractor and what is their contact information?

**Note:** Add this information to the contractor and local partner identification worksheet if not already there. If detailed information on line items costs is needed, contact the contractor and ask questions 1.1.–1.2.

### Recurrent training

*The following section should be completed by an administrator, environmental health officer, or clinical supervisor who with knowledge of trainings. For text in blue, record the responses as line items under the Recurrent Training tab of the costing spreadsheet.*

Name of Respondent:

Position/Title of Respondent:

Phone Number of Respondent:

Email of Respondent (if available):

Date of Interview:

1. What routine training is conducted for cleaning protocols and safety?
2. Are any other routine trainings conducted related to cleaning?

### Consumables

*The following section should be completed by an administrator, environmental health officer, or clinical supervisor with knowledge of sanitation facilities. For text in blue, record the responses as line items under the Consumables tab of the costing spreadsheet. Line items in this section should be consumable products (i.e., products and supplies that are consumed during normal operation; e.g., anal cleansing materials). Tools, equipment, or supplies that are re-used without being consumed over the course of normal operation are considered capital hardware and should be logged under the capital hardware tab.*

Name of Respondent:

Position/Title of Respondent:

Phone Number of Respondent:

Email of Respondent (if available):

Date of Interview:

1. What products and supplies are used for general cleaning?
2. What products and supplies are used for cleaning infectious waste and blood spills?
3. What products and supplies are used for cleaning surfaces with patient contact?
4. What products and supplies are used for disinfection and sterilization of medical devices and equipment?
5. What products and supplies are used for cleaning toilets and handwash basins?
6. What utilities are paid for operation of sluice room or other designated disinfection and sterilization area? Consider water, electric utilities, fuel needs, and other utility costs.
7. What disposable personal protective equipment are used by staff when performing cleaning duties?

### Personnel

*The following section should be completed by an administrator, environmental health officer, or clinical supervisor who supervises personnel. For text in blue, record the responses as line items under the Personnel tab of the costing spreadsheet. For all of the following questions, document the title/position of the person performing the task (e.g., laboratory technician, groundskeeper), not their specific name.*

Name of Respondent:

Position/Title of Respondent:

Phone Number of Respondent:

Email of Respondent (if available):

Date of Interview:

1. Who performs the following tasks:

**Note:** Document the title/position of the person performing the task (e.g., laboratory technician, groundskeeper), not their specific name.

- 1. General cleaning?
  2. Cleaning infectious waste and blood spills?
  3. Cleaning surfaces with patient contact?
  4. Medical device and equipment disinfection and sterilization?
  5. Cleaning toilets and handwash basins?

1. Does anyone else perform cleaning-related tasks? If so, what tasks? Who?

### Direct support

*The following section should be completed by an administrator, environmental health officer, or clinical supervisor who supervises water supply. For text in blue, record the responses as line items under the Direct Support tab of the costing spreadsheet. For all questions about people, document the title/position of the person performing the task (e.g., laboratory technician, groundskeeper), not their specific name.*

Name of Respondent:

Position/Title of Respondent:

Phone Number of Respondent:

Email of Respondent (if available):

Date of Interview:

1. Who supervises the following:
   1. Safety monitoring and audits of cleaning?
2. Does anyone else supervise cleaning-related tasks? If so, what tasks? Who?
3. Do staff involved in cleaning receive immunizations? If so, which immunizations?
4. Are any other tools, equipment, or supplies required for audits, safety monitoring, or supervision of cleaning that you have not already described? If so, please describe.

Module 6: Line item completeness evaluation

## Overview

In this module, you will evaluate the completeness of line items identified in Module 5 by comparing identified versus expected expenses. The appendix of this toolkit contains frameworks of expected expenses for each environmental service covered by this guide. There are two variants for water (piped into facility, not piped into facility) and two variants for sanitation (on-site, sewered). Select the variant that corresponds to the interview guide used in Module 5.

This module contains 1 worksheet, which should be repeated for each environmental health service:

- Worksheet 6.1: Compare identified versus expected line items

**Worksheets 6.1** contains instructions to compare line item costs identified in Module 5 to frameworks of expected costs for environmental health services included in the appendix of this toolkit. Following this comparison, you will identify data sources and a data collection plan to collect missing information.

## Instructions

This worksheet should be completed by the data collectors who completed the worksheets in Module 5. Additional input from facility stakeholders may be useful to identify data sources and develop a plan to collect missing information but is not required to perform the comparison between identified line items and frameworks of expected costs.

# Worksheet 6.1: Identified versus expected line items comparison

This worksheet should be completed separately for each environmental health service included in costing. To complete this worksheet, you will need:

- Completed worksheets from Module 4, including the corresponding spreadsheets with all line items entered.
- Framework of expected costs from the appendix that corresponds to each environmental health service. Note that two variants exist for water (piped into facility, not piped into facility) and sanitation (on-site, sewered).

Complete the following steps for each environmental health service:

- Review the spreadsheet documenting line items to ensure that data across all interviews have been entered. Cross reference the corresponding worksheet with any notes taken during the interview.
- For each cost category tab (i.e., capital hardware, capital software, etc.), compare the line items entered in the spreadsheet to the expected expenses listed in the frameworks. Highlight items which are included in both the spreadsheet and the framework.
- After you have completed the comparison, document all the expenses that are ***not*** highlighted in the table on the following page. These are expenses that are expected when providing the environmental health service, but are missing from the costing spreadsheet.
- For each missing item, discuss and document the reasons for missingness. Missingness may indicate, for example, that an item was not relevant to a particular facility or costing purpose, or that the item is relevant but was not captured in interviews.
- For each missing item, discuss whether its inclusion is important for the purpose of costing. Indicate in the table whether you will conduct additional data collection to include the item.
- For any items where follow-up data collection will be conducted, create a data collection plan. Consult Module 1 to identify potential participants, data sources, and data collection approaches. Indicate which sections, if any, of the interview guides in Module 3 will be used to collect missing information. Attach the data collection plan to this worksheet. Execute this plan to collect missing data before proceeding to Module 5.
- For items where no follow-up data collection will be conducted, justify their exclusion from cost calculations. Attach any exclusion justifications to this worksheet.

Environmental health service:

Date:

Stakeholders participating:

| Missing line item | Reason for missingness | Follow-up data collection? |
| --- | --- | --- |
|  |  |  |
|  |  |  |
|  |  |  |
|  |  |  |
|  |  |  |
|  |  |  |
|  |  |  |
|  |  |  |
|  |  |  |

Module 7: Cost data collection and calculations

## Overview

In this module, you will collect information on the costs of line items identified in Module 5, as total costs and/or as quantities and unit costs, depending on the costing purpose and level of detail required.

This module contains 3 worksheets, which should be repeated for each environmental health service:

- Worksheet 7.1: Review and revise costs data collection plan, develop data collection tools
- Worksheet 7.2: Pilot test and revise data collection tools
- Worksheet 7.3: Collect and calculate costs information

**Worksheet 7.1** contains instructions to review and revise the data collection plan generated in Module 3. You will add a section on data collection tools to support costs data collection through records review and/or interviews, as matches your costing approach. Guidance and examples to support tool development are included in this worksheet.

**Worksheet 7.2** contains instructions for pilot testing data collection tools and revising as necessary

**Worksheet 7.3** contains instructions for executing costs data collection and calculating total environmental health service delivery costs using the data collection plan and tools from Worksheet 7.1

These worksheets should be completed sequentially.

## Instructions

Use the worksheets guides in this section at least once for each environmental health service.

Worksheets 7.1 and 7.2 should be completed by stakeholders who designed the data collection plan in Module 3, as well as any data collectors who completed Modules 5 and 6. Iterate these worksheets as necessary to design appropriate data collection tools. Document all iterations and attach to this module.

Worksheet 7.3 should be completed with informants who have knowledge of the costs of line items used in environmental health service delivery. These may be service-specific experts, accountants, or procurement and logistics officers. Reference key informants identified in Module 2 Worksheets 2.1 and 2.2. If all the necessary information cannot be obtained from a single informant, you may iterate Module 2 to identify additional informants. Enter information directly into the spreadsheet, or take notes separately for later data entry. For data collection approaches using records review, attach records themselves to Worksheet 7.3, or document where records may be found for later reference if necessary. Where possible, we recommend digitizing non-electronic records. For data collection approaches using interviews and surveys, we recommend audio-recording all interviews so that recordings may be reviewed later for clarification or to extract additional detail.

# Worksheet 7.1: Review and revise costs data collection plan, develop data collection tools

### Review and revise

Review the costs data collection plan developed in Worksheet 3.2. Make any necessary revisions based on learnings from prior modules.

### Develop data collection tools

For each line item listed in your spreadsheet, you will need to determine its cost, either as a total cost or a function of quantity and unit price. The specific tool used to collect this information will depend on the costing approach and level of detail required.

For records review, you will need codebooks and decision rules to identify relevant expenses. For surveys and interviews, you will need questions to ask participants. When drafting survey questions, consider the level of detail necessary. Asking participants to, for example, estimate the average yearly salary for a cleaner will be less precise than asking each cleaner their monthly salary. Longer recall periods will be more prone to error, but shorter time periods may not capture all relevant costs or variation in costs over time.

Samples for each are provided below as guidance, but we encourage you to adapt them to your needs. If you are using a mixture of approaches, you will need to develop multiple tools

Remember that **the goal of the data collection tool is to generate information on total costs or quantities and unit prices** for all line items recorded in the costing spreadsheet. Review the costing spreadsheet before developing your tool(s).

Once you have finished your tool development, proceed to pilot testing in Worksheet 7.2.

### Sample codebook for records review

| **Code** | **Values** | **Notes / Decision rules** |
| --- | --- | --- |
| Water-related | 1 = water related, 0 = non-water related | Eligible water-related expenses are for borehole, drinking water, water at point of care, and water tower. Taps/sinks in sluice-room coded under cleaning. |
| Cost category | 1 = capital hardware, 2 = capital software, 3 = capital maintenance | Consult Table 1 for full definitions of costs categories. |
| Description | Open response | Describe the type of item (e.g., water utility bill) using descriptions in the framework of expected expenses for non-piped water. |

### Sample interview/survey questions

Capital hardware & capital software

- “When the [line item] was installed, how much did construction cost?”
- “When the [line item] was purchased, how much did it cost?”
- “When trainings and assessments were done for [line item], what was the budget for conducting them?”

Capital maintenance

- “What was the total budget last year for [line item]?”
- “How much time do [maintenance workers] spent on [maintenance task] per week/month/year? What the average salary of [maintenance worker]? In the past week/month/year, how much was spent on parts and tools for [maintenance task]?”

Recurrent training

- What was the total budget for [line item] last year?

Consumables

- On average, how many [line item] do you purchase/use in a week/month/year? How much does a [line item] cost?
- In the past week/month/year, how many [line item] did you use/purchase? How much does a [line item] cost?
- In the past week/month/year, how much did you spend on [line item]?
- In an average year, how much do you spend on [line item]?

Personnel

- What proportion of [personnel]’s duties are dedicated to [task]? What is their monthly salary?
- What proportion of [personnel]’s duties are dedicated to [task]? What the typical annual salary for this position?

Direct support

- What proportion of [personnel]’s duties are dedicated to [task]? What is their monthly salary?
- What proportion of [personnel]’s duties are dedicated to [task]? What the typical annual salary for this position?
- What is the cost of tools and supplies associated with [line item]?

# Worksheet 7.2: Pilot test and revise data collection tools

## Instructions

Pilot test your data collection plan with a subset of records or participants. For records review, select a subset of records that require coding (5–10% or enough to capture 20–30 relevant line items). For interviews and surveys, administer your questions to 3–5 participants.

Enter the data you collected into the costing spreadsheet following the instructions in Worksheet 5.3, then answer the debrief questions below. Document your responses and attach them to this worksheet.

Allocate enough time for pilot testing to administer the data collection tool, enter the data into the spreadsheet, and complete the debrief below in one sitting (2–4 hours).

### Pilot testing debrief questions

For all approaches:

- How easy or difficult is the data collection tool to apply?
- How well does the data collection tool capture necessary information?
- Does the data collection tool and resulting information fit for the costing purpose and application outlined in Worksheet 1.1?
- What worked well about the tool? What worked poorly?
- What concerns do you have about the accuracy and completeness of the resulting data?

If conducting records review:

- Are all records coders applying codes consistently?

If interviewing/surveying participants:

- Are participants able to easily answer the questions?
- Which questions, if any, cause confusion?
- Do the questions elicit the information needed?

### Revise

Based on your responses to the questions above, revise the data collection tool(s) you created in Worksheet 5.1. Keep records of all revisions and iterations to the tool, and attach them to this worksheet. If you make substantial revisions to the tool, complete this pilot testing exercise again.

# Worksheet 7.3: Collect and calculate costs information

### Execute the costing plan

Prior to executing your data collection plan, review the costing spreadsheet so you are familiar with what type of data to enter and where. If you need to adapt the spreadsheet to fit your costing purpose, do so in advance.

Execute the costing plan you have developed and revised in prior worksheets of this module. Administer your data collection tool to the data sources identified in your plan. Record the information generated on total costs and/or quantities and unit prices in the costing spreadsheet.

When administering the tool, you may record information directly into the costing spreadsheet, or take notes and enter information later. For data collection via interviews and surveys, we recommend audio-recording when possible so that recordings can be reviewed for clarification or missing information when needed.

### Calculate relevant unit costs

The costing spreadsheet included with this toolkit is designed to calculate costs in terms of total costs required to establish services (i.e., capital hardware plus capital software) and annual operations and maintenance costs (capital maintenance, recurrent training, consumables, personnel, and direct support).

Other summary measures may be useful for certain costing purposes and applications. Useful unit costs that can be calculated using information collected in Module 3 include costs per patient or healthcare provider, or costs as a percentage of overall facility budget. Consult with stakeholders to identify relevant unit costs. Some unit costs (e.g., treatment costs per kilogram of waste produced) may require additional questions to be added to the facility assessment.

### Calculate relevant financing costs

If the purpose of costing is to budget for installation of new environmental health services or major rehabilitations, you may need to include the cost of financing, such as interest or fees on loans. Financing costs will vary based on the type and amount of the loan, interest rate, and repayment period. Detailed guidance on calculating financing costs is beyond the scope of this toolkit, but users should consult with funding agencies for additional information on the specific terms of financing. Include stakeholders such as accountants, economists, or other financing experts to assist with calculating financing costs as necessary.

### Additional calculations

Depending on the costing purpose, additional calculations may be necessary. You may need to adjust for inflation, such as when budgeting for future expenses or if costs are collected from records spanning multiple prior years that no longer accurately reflect current costs. You may need to adjust for the costs of items that are donated or subsidized, where the price paid will not reflect the true cost of the item. Depending on the costing purpose, you may need to account for deprecation of goods, or annuitize costs.

Detailed guidance on these additional calculations is beyond the scope of this toolkit. However, these calculations are not specific to environmental health services, and guidance can be found in generic references on finance and costing. Several are recommended below.^1–3^ Include stakeholders such as accountants, economists, or other financing experts to assist as necessary.

### References

1. Tan-Torrees Edejer T, Baltussen R, Adam T, *et al*., eds. *Making choice in health: WHO guide to cost-effectiveness analysis.* Geneva: WHO; 2003.

2. Asian Development Bank. *Guidelines for the economic analysis of projects.* Manila: World Bank Publications; 2017.

3. Drummond MF, Sculpher MJ, Claxton K, Stoddart GL, Torrance GW. *Methods for the economic evaluation of health care programmes.* Oxford university press; 2015.

Module 8: Internal review and dissemination

## Overview

In this module, you will assess the information collected in previous models for accuracy, completeness, fitness for purpose, and develop a dissemination plan. This module is the final step.

This module contains 2 worksheets:

- Worksheet 8.1: Internal review of findings
- Worksheet 8.2: Dissemination plan

**Worksheet 8.1** contains instructions and a discussion guide to review findings internally with stakeholders who participated in modules prior to dissemination

**Worksheet 8.2** contains guidance for creating a dissemination plan for cost information and relevant information on facility context, data collection processes, and data quality

## Instructions

This module should be completed with data collectors who conducted each module, stakeholders who participated in preliminary planning of the costing purpose in Module 1, facility managers, key informants with knowledge on overall operating budgets for the facility (e.g., accountants and procurement and/or logistics officers), and subject matter experts for environmental health services.

Worksheet 8.1 is ideally completed with all participants group. This enables participants to receive and react to information in real time, receiving and discussing necessary context for a more robust evaluation. However, some participants may be briefed on group discussions and complete the worksheet individually if necessary.

# Worksheet 8.1: Internal review of findings

This worksheet should be completed separately for each environmental health service included in costing. To complete this worksheet, you will need:

- Worksheet 1.1
- All completed worksheets from Modules 4–7, including completed costing spreadsheets

### Assess completeness

Discuss and document your responses to the following questions:

**Data accuracy and completeness**

- To what extent do you believe the total costs reported in the costing spreadsheet represent the true costs of service delivery?
- To what extent do you believe the costs reported under each cost category tab of the costing spreadsheet represent the true costs of service delivery?
- What expenses are missing from costs calculations? Is collecting this information feasible?
- Any there any expenses that are double counted?
- Document and explain any instances where costs may be overestimates
- Document and explain any instances where costs may be underestimates
- How reliable are the data sources used for costs data collection? What are the limitations of the data sources used?
- Summarize the key strengths and limitations of the information collected

**Appropriateness for costing purpose**

- Are the collected data well-suited to the costing purpose identified in Worksheet 1.1?
- How similar is the facility in which data were collected to the facility/ies in which findings will be applied?
- What is the recall period for costs data?
  - For records: how many months or years do records cover?
  - For interviews: what was the recall period over which informants were asked to estimate costs? Recall period is the amount of time into the past the participant is asked to remember to answer the survey question, e.g., *“In the past year …”* versus *“In the past month …”*
  - How well does this recall period match the timeline identified in Worksheet 1.1?
- How old is the facility in which costs data were collected? How might expenses change if the facility were newer or older?

### Plan next steps

Considering your responses to the questions above, decide whether additional data collection is needed to ensure that data are complete and fit for purpose. You may iterate Modules 2-7 of this toolkit as necessary. In some cases, additional data may be desired but not available. In these instances, be sure to document and disseminate any limitations with costs findings.

Once you are satisfied that data are adequate, proceed to Worksheet 8.2 for dissemination.

# Worksheet 8.2: Dissemination plan

Costs findings should be disseminated with information on the purpose of costing and scope of costs (Module 1), facility context (Module 4), accuracy and completeness of data (Module 6), and a summary of the process used for data collection (Module 7). Document any exclusions or limitations to the data for full transparency.

The [Consolidated Health Economic Evaluation Reporting Standards (CHEERS) Statement](https://www.equator-network.org/reporting-guidelines/cheers/)^1^ contains a list of items that should be reported with costing studies. We recommend that data collectors consult the CHEERS statement to ensure that all relevant items are included in their dissemination plans.

A complete guide to developing dissemination plans is beyond the scope of this toolkit. Participating stakeholders may have their own models for dissemination plans, and partners to whom information will be disseminated may require specific formats. Additional guidance for developing dissemination plans can be found from the United States Agency for Healthcare Research and Quality^2^ and World Health Organization.^3^ Regardless of the specific model or format used for developing the dissemination plan, consult the information from Worksheet 1.1 to ensure that it is fit for purpose.

We recommend that data collectors make their data publicly available whenever possible. More widespread sharing of costs data can inform and accelerate investment in environmental health services. As cost data do not contain confidential patient information, they may be collected and shared on open-access platforms to improve available evidence and inform better decision making. However, budget information may be sensitive, so secure permissions from facility administrators, Ministries of Health, and other relevant stakeholders.

### References

1. Husereau D, Drummond M, Petrou S, *et al*. Consolidated Health Economic Evaluation Reporting Standards (CHEERS) statement. *BMJ : British Medical Journal.* 2013;346:f1049.

2. Carpenter D, Nieva V, Albaghal T, Sorra J. Dissemination Planning Tool: Exhibit A. In: Henriksen K, Battles JB, Marks ES, Lewin DI, eds. *Advances in Patient Safety: From Research to Implementation.* Vol 4. Rockville, Maryland: Agency for Healthcare Research and Quality; 2005.

3. WHO, UNICEF. *Implementation research toolkit.* World Health Organization; 2014.

Appendices: frameworks of expected expenses

These appendices contain frameworks developed by Anderson *et al*.^1^ that outline expected expenses for each environmental health service included in this toolkit. These frameworks are used to assess completeness of line item expenses identified in Module 6. Frameworks were developed through review of international guidelines for environmental health conditions in healthcare facilities in low- and middle-income countries. For additional information on framework development, see Anderson *et al*.

### References

1. Anderson DM, Cronk R, Pak E, *et al*. Development and application of tools to cost delivery of environmental health services in healthcare facilities: a financial analysis in urban Malawi. *BMC Health Serv. Res.* in press. DOI: [10.21203/rs.3.rs-72306/v1](https://doi.org/10.21203/rs.3.rs-72306/v1)

# Water—not piped into facility

| Essential outputs | Capital hardware | Capital software | Capital maintenance | Recurrent training | Consumables | Personnel | Direct support |
| --- | --- | --- | --- | --- | --- | --- | --- |
| Water source – delivery to facility | Source construction (e.g., borehole drilling) and/or  municipal water main connection  Pump infrastructure | Site assessment, engineering/architectural design, and planning for water source installation | Water source and/or water main maintenance and repairs  Pump maintenance and repairs | Water source operation and testing training | Water testing and treatment supplies  Water utility bills | Staff for water testing and treatment | Water safety planning^1^ |
| Water distribution within facility | Collection and transportation containers | n/a | n/a | Water safety and testing training | Water testing and treatment supplies  Water utility bills | Staff for transporting water to facility  Staff for water testing and treatment | Water safety planning |
| Water access at point of care | Storage containers at the point of care | n/a | Storage container maintenance and repair | Water safety and testing training | Water testing and treatment supplies  Container cleaning and restocking supplies  Water utility bills | Staff for water testing and treatment | Water safety planning |
| Water access for drinking | Water dispensers | n/a | Water dispenser maintenance and repair | Water safety and testing training | Water testing and treatment supplies  Dispenser cleaning and restocking supplies  Water utility bills  Refill jugs for water dispensers  Disposable cups | Staff for water testing and treatment  Staff for water dispenser restocking | Water safety planning |
| Emergency storage | Large storage containers (e.g. roof container or water tower) | Site assessment, engineering/architectural design, and planning for emergency storage installation | Storage container repairs and maintenance  Distribution pipes repairs and maintenance | Water safety and testing training | Water testing and treatment supplies  Container cleaning and restocking supplies  Water utility bills | Staff for water storage monitoring, flushing, and refilling | Water safety planning |
| ^1^Includes water quality testing, audits of water source safety. For a full guide, see <https://www.who.int/water_sanitation_health/water-quality/safety-planning/wsp-publications/en/> | | | | | | | |

# Water—piped into facility

| Essential outputs | Capital hardware | Capital software | Capital maintenance | Recurrent training | Consumables | Personnel | Direct support |
| --- | --- | --- | --- | --- | --- | --- | --- |
| Water source – delivery to facility | Source construction (e.g., borehole drilling) and/or municipal water main connection  Pump infrastructure | Site assessment, engineering/architectural design, and planning for water source installation | Water source and/or water main maintenance and repairs  Pump maintenance and repairs | Water source operation and testing training | Water testing and treatment supplies  Water utility bills | Staff for water testing and treatment | Water safety planning^1^ |
| Water distribution within facility | Distribution pipes  Connection points for water using infrastructure (e.g., sinks, laundry machines) | Engineering/architectural design and planning for sink and piped network installation | Distribution pipes and connection point maintenance and repairs | Water safety and testing training | Water testing and treatment supplies  Water utility bills | Staff for water testing and treatment | Water safety planning |
| Water access at point of care | Sinks/basins with connection to piped network | Engineering/architectural design and planning for sinks | Sink maintenance and repairs | Water safety and testing training | Water testing and treatment supplies  Water utility bills | Staff for water testing and treatment | Water safety planning |
| Water access for drinking | Sink with connection to piped network and/or  Water dispensers | Engineering/architectural design and planning for sinks | Sink and distribution pipe maintenance and repairs  Water dispenser maintenance and repairs | Water safety and testing training | Water testing and treatment supplies  Water utility bills  Refill jugs for water dispensers  Disposable cups | Staff for water testing and treatment  Staff for water dispenser restocking | Water safety planning |
| Emergency storage | Large storage containers (e.g. roof container or water tower) with connection to piped network | Site assessment, engineering/architectural design, and planning for emergency storage installation | Storage container maintenance and repairs  Distribution pipes maintenance and repairs | Water safety and testing training | Water testing and treatment supplies  Container cleaning and restocking supplies  Water utility bills | Staff for water storage monitoring, flushing, and refilling | Water safety planning |
| ^1^Includes water quality testing, audits of water source safety. For a full guide, see <https://www.who.int/water_sanitation_health/water-quality/safety-planning/wsp-publications/en/> | | | | | | | |

# Sanitation – non-sewered

| Essential outputs | Capital hardware | Capital software | Capital maintenance | Recurrent training | Consumables | Personnel | Direct support |
| --- | --- | --- | --- | --- | --- | --- | --- |
| Basic Sanitation Facilities^1^ | Improved toilet  Handrails, raised seats and other disability accessibility supports  Septic tank and connecting pipes | Site assessment, engineering/architectural design, and planning for toilet installation | Toilet maintenance and repairs  Septic tank maintenance and repairs  Pit/septic tank emptying services | n/a | Anal cleansing materials  Utility costs for toilet operation | Staff for restocking anal cleansing materials | Sanitary inspections |
| Menstrual hygiene management facilities^2^ | Private washing area with disposal bins with lids | Site assessment, engineering/architectural design, and planning for menstrual hygiene facility installation | Menstrual hygiene facility maintenance and repairs | n/a | Soap  Drying materials  Menstrual products (e.g., sanitary pads)  Utility costs for washing area | Staff for restocking soap and drying materials^3^  Staff for disposing menstrual products | Sanitary inspections |
| Handwashing facilities at sanitation facilities | Sink or other handwashing facility  Disposal bins for hand drying materials | Site assessment, engineering/architectural design, and planning for sinks | Sink maintenance and repairs | Hand hygiene promotion materials at defecation site | Soap  Hand drying materials | Staff for restocking soap and hand drying materials  Staff for disposing and/or recycling hand drying materials | Monitoring and inspections of hand hygiene compliance |
| ^1^ WHO guideline for number of toilets is one per 20 users for inpatient settings; at least four toilets per outpatient setting (one for staff, and for patients: one for  females, one for males and one for children) ^1^  ^2^ Menstrual hygiene management facilities may be included in sanitation facilities for women or as a separate facility | | | | | | | |

# Sanitation –sewered

| Essential outputs | Capital hardware | Capital software | Capital maintenance | Recurrent training | Consumables | Personnel | Direct support |
| --- | --- | --- | --- | --- | --- | --- | --- |
| Basic Sanitation Facilities^1^ | Improved toilet  Handrails, raised seats and other disability accessibility supports  Distribution pipes connecting to sewer mains and water source | Site assessment, engineering/architectural design, and planning for toilet installation and sewer connection | Toilet maintenance and repairs  Sewer pipe maintenance and repairs | n/a | Anal cleansing materials  Utility costs for toilet operation | Staff for restocking anal cleansing materials | Sanitary inspections |
| Menstrual hygiene management facilities^2^ | Private washing area with disposal bins with lids | Site assessment, engineering/architectural design, and planning for menstrual hygiene facility installation | Menstrual hygiene facility maintenance and repairs | n/a | Soap  Drying materials  Menstrual products (e.g., sanitary pads)  Utility costs for washing area | Staff for restocking soap and drying materials^3^  Staff for disposing menstrual products | Sanitary inspections |
| Handwashing facilities at sanitation facilities | Sink or other handwashing facility  Disposal bins for hand drying materials | Site assessment, engineering/architectural design, and planning for sinks | Sink maintenance and repairs | Hand hygiene promotion materials at defecation site | Soap  Hand drying materials | Staff for restocking soap and hand drying materials  Staff for disposing and/or recycling hand drying materials | Monitoring and inspections of hand hygiene compliance |
| ^1^ WHO guideline for number of toilets is one per 20 users for inpatient settings; at least four toilets per outpatient setting (one for staff, and for patients: one for  females, one for males and one for children) ^1^  ^2^ Menstrual hygiene management facilities may be included in sanitation facilities for women or as a separate facility | | | | | | | |

# Hygiene at points of care

| Essential outputs | Capital hardware | Capital software | Capital maintenance | Recurrent training | Consumables | Personnel | Direct support |
| --- | --- | --- | --- | --- | --- | --- | --- |
| Hand hygiene at the point of care | Sink or other handwashing facility  Dispensers for alcohol-based hand rub  Disposal bins for hand drying materials | Site assessment, engineering/architectural design, and planning for sinks and hand rub dispensers  Orientation on hand rub formulation and restocking | Sink maintenance and repairs  Hand rub dispenser maintenance and repair | Training on proper handwashing technique and handwashing promotion | Soap  Alcohol-based hand rub  Hand drying materials | Staff for restocking alcohol-based hand rub  Staff for restocking soap and hand drying materials  Staff for disposing and/or recycling hand drying materials | Monitoring and inspections of handwashing compliance  Infection prevention and control planning |
| PPE at point of care | Reusable PPE (e.g., heavy-duty aprons, protective boots) | Repairs of reusable PPE  Decontaminating, sterilization, and restocking of soiled reusable PPE | n/a | Training on proper PPE use | Disposable PPE (single-use gloves, masks, aprons) | Staff for restocking disposable PPE | Monitoring and inspections of PPE compliance  Infection prevention and control planning |

# Waste management

| Essential outputs | Capital hardware | Capital software | Capital maintenance | Recurrent training | Consumables | Personnel | Direct support |
| --- | --- | --- | --- | --- | --- | --- | --- |
| Collection, segregation, packaging, and storage | Point-of-use waste receptacles  Interim bulk storage container  Syringe/needle cutters  Storage area refrigeration units  Reusable chemical-resistant PPE (gloves, aprons, masks)  Waste weighing scale | Orientation for waste segregation and packing procedures  Site assessment, engineering/architectural design, and planning for storage area design | Maintenance and repairs to storage infrastructure  Repairs to refrigeration units | Waste segregation and safe handling training | Disposable waste containers (e.g., sharps bins, biohazard bags)  Waste labeling materials  Disposable PPE (single-use gloves, masks, aprons)  Utility costs for refrigeration units | Staff for waste collection and packing | Safety monitoring and inspections  Immunizations for waste handlers |
| Transportation:  pre- and post-treatment | Trolleys, carts, or other equipment for transport within facility  Transportation containers Transportation vehicles for off-site transport  Reusable chemical-resistant PPE (gloves, aprons, masks) | Orientation for transportation equipment and vehicle operation  Vehicle registration and licensing | Maintenance and repairs to transportation equipment  Vehicle repairs and maintenance | Sharps and hazardous waste safe handling and disposal training  Safe transport training | Disposable waste containers  Waste labeling materials  Disposable PPE (single-use gloves, masks, aprons)  Vehicle fuel | Staff for waste loading and unloading  Driver time | Vehicle insurance  Safety monitoring and inspections  Immunizations for waste handlers |
| Treatment:  autoclave^1^ | Autoclave machine and related supplies (e.g., autoclave trays)  Reusable chemical-resistant PPE (gloves, aprons, masks) | Autoclave procurement and installation costs  Orientation to autoclave operation and safety | Autoclave maintenance and repairs | Sharps and hazardous waste safe handling and disposal training | Utilities for autoclave operation  Waste labeling materials  Waste repackaging materials  Disposable PPE (single-use gloves, masks, aprons) | Staff for autoclave operation  Staff time for waste repackaging | Safety monitoring and inspections  Immunizations for waste handlers |
| Treatment: incineration^1^ | Incinerator  waste shredders; pollution control on incinerators  Reusable chemical-resistant PPE (gloves, aprons, masks, respirators) | Site assessment, engineering/architectural design, and planning for incinerator  Incinerator licensing | Treatment equipment repairs and maintenance | Sharps and hazardous waste safe handling and disposal training | Disposable waste containers  Disposable PPE (single-use gloves, masks, aprons)  Fuel costs for incinerator | Staff for incinerator operation | Incineration air quality emissions testing  Safety monitoring and inspections  Immunizations for waste handlers |
| Final disposal:  solid waste landfilling | Land costs  Landfill infrastructure (e.g., pit linings)  Pit digging equipment  On-site waste transport equipment (e.g., trucks, trolleys, etc.) | Site assessment, engineering/architectural design, and planning for landfill site  Landfill permits and licensing | Digging equipment maintenance and repair  Transport equipment maintenance and repair | Sharps and hazardous waste safe handling and disposal training | Disposable PPE (single-use gloves, masks, aprons)  Fuel for digging equipment | Staff for waste processing | Landfill licensing; compliance monitoring and inspections; immunizations for waste handlers |
| ^1^Facilities may use one or multiple methods of treatment for waste. Cost all methods used by the facility. | | | | | | | |

# Environmental cleaning

| Essential outputs | Capital hardware | Capital software | Capital maintenance | Recurrent training | Consumables | Personnel | Direct support |
| --- | --- | --- | --- | --- | --- | --- | --- |
| General Cleaning (Low level, high level, floors, walls, and doors) | Wet cleaning tools (buckets, mops, cloths)  Dry cleaning tools (brooms, dustpans, cloths)  Reusable chemical-resistant PPE (gloves, aprons) | Orientation to cleaning protocols for newly hired staff | n/a | Refresher trainings on cleaning protocols and safety | Disposable PPE (single-use gloves, masks, aprons)  Disposable cloths  Cleaning chemicals (detergents, soaps) | Staff time for cleaning | Safety monitoring and inspections  Immunizations for cleaners |
| Cleaning infectious waste/blood spills | Wet cleaning tools (buckets, mops, cloths)  Dry cleaning tools (brooms, dustpans, cloths)  Reusable chemical-resistant PPE (gloves, aprons, face shields) | Orientation to cleaning protocols for newly hired staff  Orientation to hazardous waste safety for newly hired staff | n/a | Refresher trainings on cleaning protocols  Refresher trainings on hazardous waste safety | Disposable PPE (single-use gloves, masks, aprons)  Disposable cloths, absorbent material  Cleaning chemicals (detergents, soaps, antiseptics) | Staff time for cleaning | Safety monitoring and inspections  Immunizations for cleaners |
| Cleaning surfaces with patient contact (e.g., mattresses, delivery beds) | Wet cleaning tools (buckets, mops, cloths)  Dry cleaning tools (brooms, dustpans, cloths)  Reusable chemical-resistant PPE (gloves, aprons) | Orientation to cleaning protocols for newly hired staff  Orientation to hazardous waste safety for newly hired staff | n/a | Refresher trainings on cleaning protocols  Refresher trainings on hazardous waste safety | Disposable PPE (single-use gloves, masks, aprons)  Disposable cloths, absorbent material  Cleaning chemicals (detergents, soaps, antiseptics) | Staff time for cleaning | Safety monitoring and inspections  Immunizations for cleaners |
| Medical device/equipment disinfection and sterilization | Washing and decontamination area with wastewater disposal (e.g., sluice room)  Sterilization equipment (steam sterilizer, low temp sterilizers, or chemical sterilizers)  Reusable chemical-resistant PPE (gloves, aprons) | Orientation to cleaning protocols for newly hired staff  Orientation to hazardous waste safety for newly hired staff  Site assessment, engineering/architectural design, and planning for decontamination area design  Sterilization equipment procurement and installation costs  Orientation to sterilization equipment operation and safety | Wastewater disposal area maintenance and repairs  Sterilization equipment maintenance and repairs | Refresher trainings on cleaning protocols  Refresher trainings on hazardous waste safety | Disposable PPE (single-use gloves, masks, aprons)  Cleaning chemicals (detergents, soaps, antiseptics)  Packaging and wrapping for medical equipment  Labeling materials  Utilities for decontamination and sterilization equipment operation | Staff time for device packing & initial decontamination Staff time for sterilization equipment operation  Staff time for device repacking and restocking | Safety monitoring and inspections  Immunizations for cleaners |
| Cleaning toilets and handwash basins | Wet cleaning tools (buckets, mops, cloths)  Dry cleaning tools (brooms, dustpans, cloths)  Reusable chemical-resistant PPE (gloves, aprons, face shields) | Orientation to cleaning protocols for newly hired staff  Orientation to hazardous waste safety for newly hired staff | n/a | Refresher trainings on cleaning protocols  Refresher trainings on hazardous waste safety | Disposable PPE (single-use gloves, masks, aprons)  Disposable cloths, absorbent material  Cleaning chemicals (detergents, soaps, antiseptics) | Staff time for cleaning | Safety monitoring and inspections  Immunizations for cleaners |
